# Using Group II Introns for Attenuating the *In Vitro* and *In Vivo* Expression of a Homing Endonuclease

**DOI:** 10.1371/journal.pone.0150097

**Published:** 2016-02-24

**Authors:** Tuhin Kumar Guha, Georg Hausner

**Affiliations:** Department of Microbiology, University of Manitoba, Winnipeg, Canada; Institut de Genetique et Microbiologie, FRANCE

## Abstract

In *Chaetomium thermophilum* (DSM 1495) within the mitochondrial DNA (mtDNA) small ribosomal subunit (*rns*) gene a group IIA1 intron interrupts an open reading frame (ORF) encoded within a group I intron (mS1247). This arrangement offers the opportunity to examine if the nested group II intron could be utilized as a regulatory element for the expression of the homing endonuclease (HEase). Constructs were generated where the codon-optimized ORF was interrupted with either the native group IIA1 intron or a group IIB type intron. This study showed that the expression of the HEase (*in vivo*) in *Escherichia coli* can be regulated by manipulating the splicing efficiency of the HEase ORF-embedded group II introns. Exogenous magnesium chloride (MgCl_2_) stimulated the expression of a functional HEase but the addition of cobalt chloride (CoCl_2_) to growth media antagonized the expression of HEase activity. Ultimately the ability to attenuate HEase activity might be useful in precision genome engineering, minimizing off target activities, or where pathways have to be altered during a specific growth phase.

## Introduction

Homing endonucleases (HEases) are site-specific DNA cleaving enzymes that are encoded by homing endonuclease genes (HEGs) which are frequently found embedded within archaeal introns, composite mobile genetic elements such as group I introns, group II introns, inteins [1–5] and sometimes HEGs are freestanding [5–9]. The LAGLIDADG family (LAG HEase) are frequently encoded within fungal mitochondrial group I introns [10, 11] and HEases in general recognize long asymmetrical 12–40 bp of DNA sequences as their target sites and cleave in a manner that generates four nucleotide 3′-overhangs [6–8]. As LAG HEases require long DNA target sequences they cut infrequently within a genome [8] and this feature has been utilized for various applications in genome editing as it relates to agriculture [12], population control of disease vectors [13–15], and human health [16–19]. In this study we are testing the possibility of utilizing mitochondrial group II intron sequences as an on/off “switch” system that provides the opportunity for temporal control of HEase activity in *Escherichia coli*.

Previously a molecular switch was developed that controlled the endonuclease activity of PI-SceI *in vitro*; here two cysteine amino acid residue pairs were separately inserted into the HEase DNA binding loops to allow for disulfide bond formation that lock the endonuclease into a nonproductive conformation [19]. This essentially is a redox switch and the activity of the protein could be controlled by adding or removing a reducing agent [19]. Other strategies suggested for regulating endonuclease activity included manipulating metal ion cofactors (such as Mg^+2^) or developing temperature sensitive versions of HEases [20]. However, in order for a true on/off “switch” it was suggested that manipulating cellular concentrations of ion cofactors might be difficult and using different temperatures might pose a problem with some cell lines or some temperature sensitive HEases once misfolded could not be reactivated (i.e. refolded) [19]. Redox switches as developed for PI-SceI have potential for *in vitro* applications but are not practical for *in vivo* applications as the targeted cell would probably suffer damage if oxidizing conditions were applied.

Recently we characterized a twintron-like arrangement (or ‘nested’ intron) at position S1247 in the mitochondrial DNA (mtDNA) small ribosomal subunit (*rns*) gene of *Chaetomium thermophilum* var. *thermophilum* La Touche (strain DSM 1495). The external intron encodes a double-motif LAG HEase ORF (I-CthI) which is interrupted by an internal ORF-less group IIA1 intron [21]. The group IIA1 intron was shown by *in vitro* self-splicing experiments to be excised and thus generating a transcript where the LAG HEase ORF is contiguous and could allow for the expression of a functional endonuclease [22]. Splicing of the internal group II intron could be a regulatory step that allows for the maturation of the HEase transcript and translation of the open reading frame. Splicing of group II introns requires the intron RNA to assume a splicing competent tertiary fold that includes interactions between intron and flanking exon sequences. Folding of the intron RNA is in part facilitated by base pair complementarities [23]. In addition various intron and/or host genome encoded factors tend to assist *in vivo* RNA folding and splicing of group II intron from transcripts [23, 24]. Group II intron RNAs are ribozymes composed of six helical regions, referred to as domains I through VI, emerging from a central wheel and these domains interact to form a conserved tertiary structure that brings together distant sequences to form an active site. The active site binds the splice sites and the branch-point nucleotide residue and uses bound Mg^+2^ ions to activate the appropriate bonds for catalysis [25, 26]. Terbium cleavage assay determined domain V (DV) to be an important region of the active site as it contains the catalytic triad AGC and an AY bulge, both of which bind Mg^+2^ [27, 28]. Experimental results from phosphorothioate substitutions at the splice sites suggested that group II introns either use separate active sites with different Mg^+2^ ions to catalyze the two splicing (transesterification) steps or a single active site which is rearranged between the steps [29, 30]. Moreover, X-ray crystallographic structure of the catalytic core of an *Oceanobacillus iheyensis* IIC intron revealed that two helices of DV are bent to bring the AC bulge near the CGC triad, thus juxtaposing the phosphate backbones of the most conserved DV sequences, rather than stacking coaxially [31]. Nine potential Mg^+2^ binding sites were assigned in or near DV for this intron [32]. Hence, a potential trigger for splicing might involve the availability of Mg^+2^ inside *E*.*coli* cells and this property has been applied in this research to develop a mechanism for the *in vivo* regulation of HEase (I-CthI) activity.

In order to evaluate if group II intron sequences could be manipulated as regulatory elements that could prevent or at least attenuate HEase expression constructs have been generated where a pET28b (+) expression vector contains the I-CthI sequence that is interrupted by its native group IIA1 intron sequence [21]. The HEase sequence was optimized for expression in *E*. *coli* and for maintaining the intron binding sequences (IBS1 and IBS2) which are located upstream of the intron insertion site. The IBS elements are needed for splicing as they interact with the corresponding exon binding sequences (EBS1 and EBS2) present within the intron in part to establish a splicing competent fold [32]. A second construct was designed where the native IIA1 intron was replaced by a mitochondrial group IIB intron (no ORF) [rI1 of *Scenedesmus obliquus*] along with its corresponding IBS1 sequence [33].

It has been suggested that the *in vivo* control of HEase activity would allow for more precise temporal inactivation or modification of genes [34, 35]; for example, to shift metabolic processes during a particular growth phase of the bacteria that are being manipulated for the production of certain metabolites or proteins [36].

## Materials and Methods

### Design of the *Escherichia coli* expression vectors and substrate

An expression plasmid with the codon-optimized version of the I-CthI ORF along with its native internal group IIA1 intron (GenBank accession number: JX139037.1) was synthesized by Genscript (New Jersey, USA). However, the IBS1 and IBS2 sequences which are upstream of the intron insertion site (IBS1: 5′ TGTTTT 3′, IBS2: 5′ TTTAAT 3′) and thus located within the HEase ORF were not modified in order to maintain the splicing potential of the group IIA1 intron. The synthesized ORF sequence (1722 bp) was inserted into the pET28b (+) plasmid as a NheI/BamHI fragment. The vector provides the ORF with an N-terminal 6X histidine-tag; this construct was named I-CthI-[IIA1]-pET28b (+).

**Fig 1:**
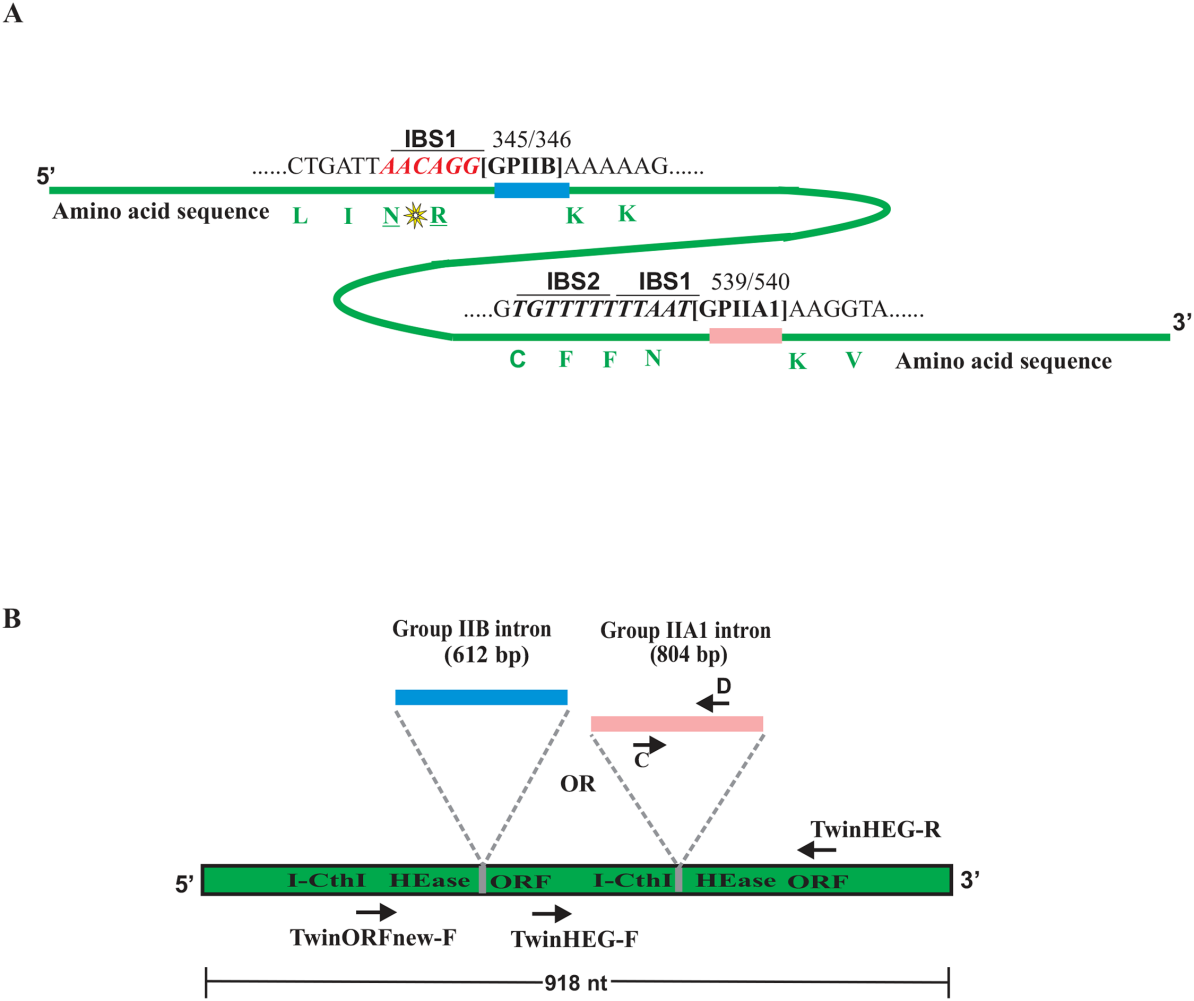
**(A) Homing endonuclease ORF and location of introns.** Schematic representation (not drawn to scale) for the location of the native group IIA1 intron (GPIIA1, shown in pink) and the non-native group IIB intron (GPIIB, shown in blue) within the I-CthI HEase ORF sequence (shown in green). Number 539 and 540 represent the exact location (insertion site) of the GPIIA1 in the ORF sequence. The IBS1 and the IBS2 elements are in italics (underlined), both are located upstream from the intron insertion site. The corresponding amino acids (marked in green) have been indicated to their respective codons. In another construct, GPIIB was inserted in the I-CthI HEase ORF sequence at position 345/346 that allowed for maintaining the IBS/EBS interactions with minimal modification of the HEase coding sequence (see text for details). The conservative amino acid substitution (indicated with a yellow star) between Arginine (R) and Asparagine (N) residues has been introduced to form the correct IBS1 (marked in red, italics and underlined) for supporting GPIIB splicing. The corresponding amino acids (marked in green) have been indicated to their respective codons. **(B) Overview of primer location for RT-PCR.** Diagram (not drawn to scale) showing the relative location of the RT-PCR primers within I-CthI HEase ORF sequence utilized for detecting splicing products during the *in vivo* RNA splicing assay.

In order to expand the concept of the “intron based on switch” as a potential regulatory element for HEase expression, a non-native group IIB intron (rI1) from *Scenedesmus obliquus* (GenBank accession number X17375.2) [37] was inserted in a suitable position within the I-CthI ORF. The intron was inserted at a location that allowed for maintaining the IBS/EBS interactions with minimal modification of the HEase coding sequence; fortuitously for the rI1 intron only the IBS1 sequence is essential for splicing [33]. Prior to the insertion of this intron, a suitable region in the HEase ORF sequence was located, that matched the required IBS1 (*aac* coding for arginine and *agg* coding for asparagine) element for this group IIB intron. However, the presence of this sequence at the carboxyl terminal of the HEase ORF made it less suitable. An alternative approach was undertaken. A sequence located further upstream, *cgcaac* encoding asparagine and arginine, was rearranged to *aaccgc* essentially introducing two conservative amino acid replacements. The *cgc* nucleotides were further modified to *agg* which did not change the amino acid composition of the protein (Fig 1A). The codon-optimized ORF (1530 bp) including the intron was synthesized and inserted as a NdeI/BamHI fragment into the pET28b (+) plasmid (same as for the native intron). The construct was named I-CthI-[IIB]-pET28b (+). Both of the constructs were transformed into *E*. *coli* BL21 (λDE3) (chemically competent cells, New England Biolab, MA, USA) for testing the *in vivo* group II intron splicing competency and for additional biochemical studies.

A substrate plasmid was previously constructed in order to evaluate the activity of I-CthI [22]. Briefly, a segment of the *rns* sequence (GenBank accession number: JN007486.1) flanking the mS1247 nested intron was synthesized by Genscript (New Jersey, USA) and cloned in pUC57 vector as an EcoRV fragment. This substrate plasmid was named Cth-*rns*.pUC57 and the overall size of the construct is 3.1 kb. Most of the chemicals and the reagents used in this study were purchased from Thermo Fisher Scientific unless otherwise mentioned in the text.

### *In vivo* RNA splicing assay

Reverse Transcriptase PCR (RT-PCR) was employed to examine *in vivo* splicing intermediates for the HEase ORF group II introns. Here the HEase gene derived transcripts were studied to verify if splicing occurred and if splicing in *E*. *coli* maintained the expected intron/exon junctions. One hundred μL of chemical competent *E*. *coli* BL21 (λDE3) were transformed with either I-CthI-[IIA1]-pET28b (+) or the empty pET28b (+) vector and inoculated in 10 mL of Luria Bertani (LB; peptone 10 g/L, yeast extract 5 g/L and NaCl 5 g/L; pH 7.0) media supplemented with 100 μg/mL kanamycin and 0.25% w/v glucose and incubated overnight (O/N) with agitation at 37°C. Five hundred μL from the O/N culture was used to inoculate 50 mL of LB medium supplemented with kanamycin (kan) and glucose (as described above). Additionally, each of the culture flasks was supplemented with either 1 mM, 5 mM, 10 mM or 20 mM magnesium chloride (MgCl_2_). Initially, exogenous MgCl_2_ concentrations up to 100 mM were tested. Even though such high concentrations of MgCl_2_ was not detrimental to the bacterial cells (as evident from checking O.D._600_), there was no appreciable difference in the splicing activity as compared to the culture containing 20 mM MgCl_2_. Therefore, MgCl_2_ concentrations up to 20 mM were considered for further studies. A culture flask with no exogenously added MgCl_2_ was used as the negative control. A second set of negative controls consisted of cultures either: a) 10 μM of cobalt chloride (CoCl_2_) was added to the LB media or b) the LB media was supplemented with both 10 μM CoCl_2_ and 5 mM MgCl_2_. The cultures were grown at 37°C with agitation till the O.D._600_ reached 0.65. Ten mL of the bacterial cells from each of the above cultures were centrifuged for 3 minutes at 7000 rpm. The cells were lysed and RNA was extracted using the GENEzol TriRNA Pure kit (FroggaBio, North York, Ontario) following the manufacturer’s protocol. To ensure complete removal of any contaminating DNA, the RNA was treated with 2 units of DNaseI (Fermentas, Ottawa, Canada) and incubated at 37°C for 15 minutes; the reaction was stopped by adding 1 μL EDTA (50 mM) followed by 10 minute incubation at 65°C. Furthermore, in order to confirm the elimination of the DNA, 2 μL of the reaction mixture was applied to perform a standard PCR reaction using the forward primer TwinHEG-F (5′-ATGTGGTTATCCCGCATTTCG-3′) and the reverse primer TwinHEG-R (5′-TTGAAGTTTTCGTTCTTGATGCC-3′; Fig 1B).

The ThermoScript RT-PCR system (Life Technologies) was used to make cDNA from the RNA extracted from bacterial cells grown in the presence of various concentrations of MgCl_2_. Briefly, for the first strand synthesis a 20 μL reaction mix was prepared containing 1 μg RNA, 0.5 mM of the reverse primer TwinHEG-R, 0.1 M DTT, 4 μL of 5X cDNA synthesis buffer, 1 mM of each dNTP, 40 units RNase-OUT (Life Technologies) and 15 units of Thermoscript reverse transcriptase. Reverse transcription was performed at 55°C for 1 hour and stopped by heating the reaction mixture to 85°C for 10 minutes. Finally, 1 μL of RNase H (2 units) was added to the reaction mixture followed by incubation at 37°C for 20 minutes.

In order to characterize the expression vector derived HEase transcripts several primers were designed to recover potential splicing intermediates. The forward primer TwinHEG-F and the reverse primer TwinHEG-R (see above) are based on the codon-optimized ORF upstream (290 nucleotides) and downstream sequences (248 nucleotides) with regards to the group IIA1 intron insertion site (Fig 1B). Primer‘C’ (5′- ACAGCATGCAGCAAAAGCGG-3′) and primer‘D’ (5′-TGTATAACATCTCAGCCGACTGCC-3′) [22] were used to amplify a 400 bp segment of the internal group IIA1 intron and this segment served as the positive control for detecting the presence of the group IIA1. In order to examine the splicing potential and splicing intermediates for constructs featuring the rI1 intron, the same protocol was applied as above. Since the location of the group IIB intron within the ORF is further upstream compared to the native intron, one additional forward primer was designed (TwinORFnew-F: 5′-ATAAGAACGATCTGGAAGTCCTGC-3′) in order to capture the splicing products that involved the rl1 intron. The reverse primer (TwinHEG-R) remained the same for the group IIB intron splicing analysis. The RT-PCR amplicons obtained were excised from the agarose gel and purified using the Gel/PCR DNA fragments Extraction Kit (FroggaBio, North York, Ontario). The gel extracted DNA fragments were sent to the MICB DNA sequencing facility (University of Manitoba, Cancer Care, McDermot Avenue, Winnipeg, Canada) for Sanger cycle sequencing utilizing the primers used for obtaining the amplicons (Fig 1B).

### *In vitro* and *in vivo* protein expression and purification

For both types of constructs I-CthI-[IIA1]-pET28b (+) and I-CthI-[IIB]-pET28b (+), protein expression was first evaluated with an *in vitro* translation assay. RNA extracted from *E*.*coli* BL21 cells grown under different Mg^+2^ concentrations were further subjected to *in vitro* translation. The PURExpress *In Vitro* Protein Synthesis Kit (New England Biolab, MA, USA) which is a cell-free transcription/translation system was utilized to assess if the proteins can be expressed in an “*E*. *coli*” environment. Although the PURExpress kit is designed for coupled transcription and translation from an expression construct, direct translation from an mRNA template is also possible provided purified RNA (1–5 μg) is added to the reaction mixture, albeit a proper ribosome binding site must be present for efficient translation. After three hours of incubation at 37°C, 2.5 μL of the reaction sample was mixed with 2.5 μL of the 2x protein loading dye [65.8 mM Tris-HCl, pH 6.8, 26.3% (w/v) glycerol, 2.1% SDS, 0.01% Bromophenol blue] and subjected to 12.5% SDS PAGE (BioRad, Mississauga, Ontario). The gel was stained in Coomasie Brilliant Blue (Roche, Mississauga, Ontario) and analyzed for the presence of the desired protein at 29 kDa.

For *in vivo* protein overexpression in *E*. *coli* and protein purification, the same protocols were applied for both types of constructs I-CthI-[IIA1]-pET28b (+) and I-CthI-[IIB]-pET28b (+). In order to check for the expression of the HEase protein *in vivo*, the remaining 40 mL of culture (first 10 mL from each 50 mL culture were removed for RNA extraction to perform *in vivo* RNA splicing assay; see above) was induced with Isopropyl β-D-1-thiogalactopyranoside (IPTG; Life Technologies) to a final concentration of 0.5 mM when the O.D._600_ of the cells reached 0.65. The cultures were then shifted to 28°C and incubated with agitation for 4 hours. Cells were resuspended in the lysis buffer [50 mM Tris-HCl (pH 8.0), 100 mM NaCl, 10% (w/v) glycerol, 6 mM β-mercaptoethanol] at a ratio of 5 mL of buffer to 1 g of cells (wet weight) and sonicated in short pulses of 15 seconds using the Sonic Dismembrator model 300 (Thermo Fisher Scientific). Eight μg of crude lysate from each of the induced samples were subjected to 12.5% SDS PAGE. Gels were stained in Coomasie Brilliant Blue (Roche, Mississauga, Ontario) and analyzed for the presence of the desired protein band. Ni-NTA super flow column (Qiagen, Toronto) was used to purify the protein following the methods described previously [22].

### *In vitro* endonuclease assay

The I-CthI HEases as expressed from constructs containing either the native group IIA1 intron or the rI1 group IIB intron were evaluated for activity by performing *in vitro* endonuclease assays as previously described [22]. Briefly 1 μg of substrate plasmid Cth-*rns*.pUC57 was treated with 8 μL of purified HEase protein (3 mg/mL) in the endonuclease reaction buffer [50 mM Tris-HCl (pH 8.0), 10 mM MgCl_2,_ 100 mM NaCl] and the reactions were stopped at specific time intervals 0, 30, 60, 90 and 120 minutes. The digested products were resolved via agarose gel electrophoresis. Two non-substrate sequences were also challenged with the HEase preparations to evaluate the specificity and purity of the HEases extracts. A *C*. *thermophilum rns* segment containing the mS1247 nested intron plus flanking exon sequences (GenBank accession number: JN007486.1) cloned in the pUC57 vector, and a fragment of the cytochrome oxidase (*cox*) gene *from Annulohypoxylon stygium* (GenBank accession number: NC_023117.1) cloned into the pUC57 vector served as negative controls. These non-substrates (1 μg) were challenged with the same concentration of the HEase protein and incubated for two hours at 37°C. In one set of reactions, in order to rule out the possibility that addition of CoCl_2_ has any inhibitory effect on the I-CthI HEase’s activity, 10 μM of CoCl_2_ was added to the *in vitro* endonuclease reaction buffer and the above protocol was followed. Moreover, to test the effect of the activity of the protein in the presence of only CoCl_2_ (and not in combination with MgCl_2_), the protein was incubated with the substrate while 10 μM of CoCl_2_ was added to the endonuclease reaction buffer minus 10 mM MgCl_2_.

### Cleavage site mapping assay

The Cth-*rns*.pUC57 substrate construct was incubated with the I-CthI protein expressed from both types of intron containing constructs. The linearized substrate plasmids were recovered and treated with T4 DNA polymerase (Life Technologies) in order to remove the expected four nucleotide 3′-overhangs. Treated DNA fragments were religated with Quick ligase (New England Biolab, MA, USA) following the manufacturer’s protocol. After religation these plasmids were transformed into *E*. *coli* and eventually reisolated and sequenced. The cleavage mapping assay was performed as previously described [38, 39] and the cleavage site can be designated by noting which nucleotides have been removed during the T4 DNA polymerase treatment from the substrate plasmid.

### Evaluating the role of MgCl_2_ in stimulating HEase expression

**Fig 2:**
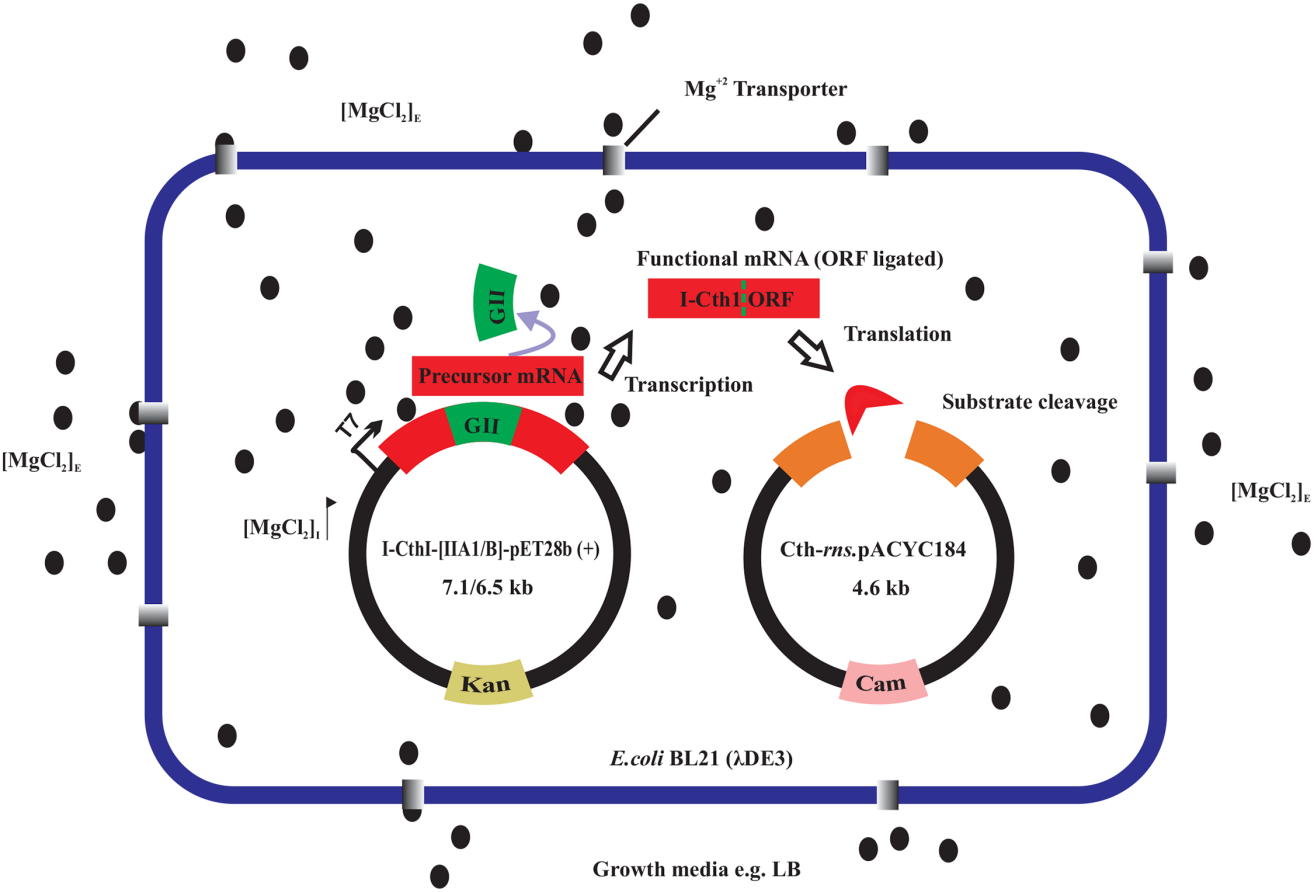
Impact of MgCl_2_ on splicing and the expression of a homing endonuclease. An *in vivo* endonuclease assay was established where two compatible plasmids were maintained in *E*. *coli* BL21 (λDE3) based on antibiotic selection [kanamycin (kan) and chloramphenicol (cam)]. Cells were grown in absence and presence of either 5 mM or 10 mM added MgCl_2_ (E = external) and induced with 0.5 mM IPTG (O.D._600_ = 0.56). The internal concentration (I) of MgCl_2_ increases probably due to the activities of magnesium transporters. Free Mg^+2^ ions are available to bind to the catalytic center of the group II intron [domain V (not shown)] and initiate efficient splicing and religation of the ORF. The expressed HEase cleaves the target site in the substrate plasmid resulting in the loss of cam resistance marker. Cells grown in the absence of MgCl_2_ fail to splice out the internal group II thus yielding non-functional HEase thereby resulting in the maintenance of the substrate plasmid and hence the survival of colonies on cam plates. The *E*.*coli* genome is not shown for simplicity.

In order to evaluate if intron splicing could be manipulated as a potential “on switch”, the addition of exogenous Mg^+2^ was investigated with regards to expression of the HEases. An *in vivo* endonuclease assay was established to evaluate the expression of functional HEases at various Mg^+2^ concentrations. Here two compatible plasmids were maintained in *E*.*coli* BL21 (λDE3) based on antibiotic selection [kanamycin (kan) and chloramphenicol (cam)]. The I-CthI-[IIA1]-pET28b (+)—kan (ColE1 origin of replication) construct (7.1 kb) allowed for the expression of the HEase ORF and a second plasmid with cam and the appropriate HEase target site sequence served as the substrate plasmid. For constructing the substrate plasmid, the Cth-*rns*.pUC57 was digested with BamHI (Life Technologies) and XbaI (Life Technologies) and a 469 bp containing the *rns* segment with the HEase target site was cloned in the pACYC184 plasmid [ATCC 37033 (American Type Culture Collection, Manassas, VA, USA; p15A origin of replication].The substrate plasmid was named Cth-*rns*.pACYC184—cam (4.6 kb). Excision of the intron would permit the expression of an active HEase from the I-CthI-[IIA1]-pET28b (+)—kan plasmid and this could be detected by plating cells on various media with different antibiotics, ultimately the presence of the HEase should lead to the loss of the Cth-*rns*.pACYC184 containing the cam resistance marker (Fig 2).

For the *in vivo* endonuclease assay, co-transformed *E*. *coli* BL21 (λDE3) cells were grown overnight in duplicates in culture tubes containing 5 mL LB media plus the appropriate antibiotics. One percent glucose was added to the media containing the HEase-co-transformed construct to prevent leaky expression from the T7 promoter. A 0.5 mL aliquot from the 5 mL O/N cultures was used to inoculate 50 mL LB broth cultures supplemented with 100 μg/mL kan, 60 μg/mL cam, 1% glucose and 5 mM MgCl_2_. For convention, we refer to this culture flask as ‘LB+Mg^+2^’. Another culture flask designated ‘LB’ which contained no added MgCl_2_ was inoculated with the same amount of O/N culture and this served as the negative control for this experiment. The cells were grown at 37°C with vigorous shaking (210 rpm) and the cultures were either induced with 0.5 mM IPTG when the O.D._600_ reached ~ 0.56 or not induced. The cultures were then shifted to 28°C for the production of the HEase. After 4 hours, both the induced and uninduced cultures from ‘LB+Mg^+2^’ and ‘LB’ were diluted to 10^−6^ and 100 μL of the diluted cultures were plated on LB agar plates containing 60 μg/mL cam (done in triplicate). Plates were incubated at 37°C until the colonies were clearly visible; colonies were counted approximately after 18 hours of incubation. For this experiment, three technical and two biological replicates were performed. In addition, to ensure that proteins expressed by the empty pET28b (+) vector (without HEase ORF intron containing construct) were not involved in the endonuclease activity, 50 ng of the vector was also co-transformed along with Cth-*rns*.pACYC184 into 100 μL of chemically competent *E*.*coli* BL21 (λDE3) cells and the above protocol was followed. To evaluate the effect of 5 mM exogenous MgCl_2_ on the *in vivo* splicing potential of the rI1 group IIB intron, the I-CthI-[IIB]-pET28b (+)—kan construct was cotransformed with the substrate plasmid and the *in vivo* endonuclease protocol was performed as described above.

In order to antagonize the stimulatory effect of MgCl_2_ on splicing of group II introns, 10 μM of cobaltous chloride (CoCl_2_) was added to the culture media along with 5 mM MgCl_2._ It has been previously shown that CoCl_2_ perturbs the import of Mg^+2^ in *E*.*coli* cells [40–42]. Moreover, in order to negate the possibility that CoCl_2_ can promote splicing of the group IIA1 or group IIB, *E*.*coli* BL21 (λDE3) cells containing each of the constructs I-CthI-[IIA1]-pET28b (+) and I-CthI-[IIB]-pET28b (+) were exposed to 10 μM of CoCl_2_ in the culture media. The cultures containing both the salts (MgCl_2_ and CoCl_2_) as well as CoCl_2_ alone were either uninduced or induced with 0.5 mM IPTG when the O.D._600_ reached ~ 0.5. The cultures were further incubated at 28°C for the production of the HEase. After 4 hours, the cultures were diluted to 10^−6^ and 100 μL of the diluted cultures were plated on each of the LB agar cam selection plates (done in triplicate). The plate assays were performed as described above in order to evaluate the splicing of the internal group II introns in the presence of CoCl_2._ For statistical analysis, unpaired student’s t test was performed to determine the significance of the results obtained. Graphpad Prism 6.01 statistical analysis software was used to calculate the Student’s t test and the respective bar graphs were drawn using the same software.

## Results

### Exogenous Mg^+2^ induces *in vivo* splicing of group IIA1 and group IIB introns

**Fig 3:**
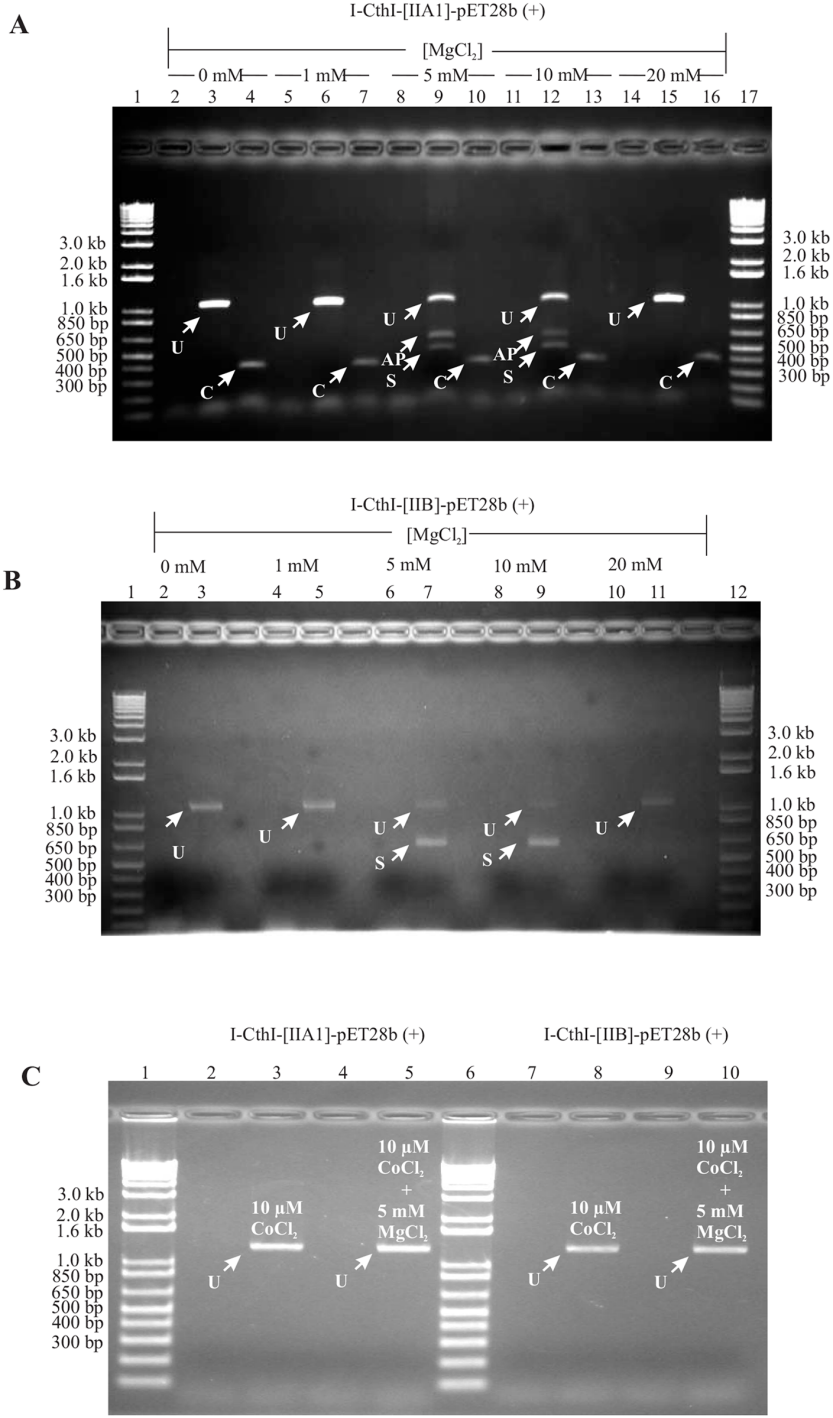
**(A) A mtDNA group IIA1 intron can splice in *E*. *coli*.** A 1% agarose gel showing the RT-PCR results (Primers: TwinHEG-F/R) for *in vivo* group IIA1 intron splicing under various concentrations of external MgCl_2_ in the culture media. Lanes 2, 5, 8, 11, 14 show the results for standard PCR (TwinHEG-F/R) on RNA samples testing for the absence of genomic DNA prior to cDNA synthesis. Lane 3, 6 (see white arrows) and 15 show the PCR product (~1.1 kb) that represents the unsliced transcript (U) in the absence (0 mM), presence of low (1 mM) or high (20 mM) concentration of MgCl_2_ in the culture media respectively. Lanes 9 and 12 (see white arrows) show the PCR product (556 bp) that indicates splicing occurred (S) and possible alternative products (AP) are also indicated in lanes. Lanes 4, 7, 10, 13 and 16 (see white arrows) are positive controls (Primers C and D) that show the presence of the internal group IIA1 (indicated by C; 400 bp) in all the samples examined. Lane 1 and 17 contain the 1kb plus DNA ladder (Life Technologies). For more information on primers see text and Fig 1B. **(B) A mtDNA group IIB intron can splice in *E*. *coli*.** A 1% agarose gel showing the RT-PCR results (Primers: TwinORFnew-F/TwinHEG-R) for *in vivo* group IIB intron splicing under various concentrations of external MgCl_2_ in the culture media. Lanes 2, 4, 6, 8 and 10 (see white arrows) show the results for standard PCR (Primers: TwinORFnew-F/TwinHEG-R) on RNA samples testing for the absence of genomic DNA prior to cDNA synthesis. Lanes 3, 5 and 11 (see white arrows) show the PCR product (~1.2 kb) that represent unspliced transcripts (U) in the absence (0 mM), presence of low (1 mM) or high (20 mM) concentration of MgCl_2_ in the culture media respectively. Lanes 7 and 9 (see white arrows) show a PCR product that represents spliced (S) transcripts (658 bp) in the presence of 5 mM and 10 mM MgCl_2_ in the culture media. Lane 1 and 12 contain 1kb plus DNA ladder (Life Technologies). **(C) Effect of CoCl**_**2**_ **and/or MgCl**_**2**_ **on intron splicing**. A 1% agarose gel showing the RT-PCR results for *in vivo* splicing of group IIA1 intron and group IIB intron when 10 μM CoCl_2_ alone or 10 μM CoCl_2_ in combination with 5 mM MgCl_2_ were added to the LB growth media. Lanes 2 and 4 show the results for standard PCR on RNA samples testing for the absence of genomic DNA prior to cDNA synthesis. Lane 3 and 5 show that splicing cannot be detected (see band marked with U) when I-CthI-[IIA1]-pET28b (+) [BL21] was grown with the addition of 10 μM CoCl_2_ in one LB culture media and combination of 10 μM CoCl_2_ and 5 mM MgCl_2_ in the other LB media respectively. For the second construct I-CthI-[IIB]-pET28b (+) [BL21], lanes 7 and 9 represent the results for standard PCR on RNA samples testing for the absence of genomic DNA prior to cDNA synthesis. Lanes 8 and 10 show that splicing cannot be detected (see band marked with U) when I-CthI-[IIB]-pET28b (+) [BL21] was grown with the addition of 10 μM CoCl_2_ in one LB culture media and combination of 10 μM CoCl_2_ and 5 mM MgCl_2_ in the other LB media respectively. Lanes 1 and 6 represent 1kb plus DNA ladder (Life Technologies).

To demonstrate the splicing competency of group IIA1 and IIB intron in the presence of various added concentrations of exogenous Mg^+2^, RNA was extracted from *E*. *coli* BL21 cells containing the constructs I-CthI-[IIA1]-pET28b (+) or I-CthI-[IIB]-pET28b (+). The splicing reaction products for the native intron were recovered with RT-PCR utilizing primers TwinHEG-F and TwinHEG-R. Among the observed cDNAs obtained from RNA extracted from bacterial cells grown in the presence of various concentrations, only cells grown at 5 mM and 10 mM MgCl_2_ showed evidence of alternate products. A PCR product near the 500 bp marker, the expected size (538 bp) for cDNAs from transcripts where the group IIA1 intron spliced out, was further investigated (Fig 3A). Bacterial cells grown in either lower (0 mM, 1 mM) or higher (20 mM) MgCl_2_ did not show any evidence for splicing and only the full length unspliced PCR product (1296 bp) was recovered. A 400 bp PCR product was observed when internal group IIA1 intron specific primers (‘C’ and ‘D’) were used and this served as the positive control showing the presence of the intron in all the samples examined. PCR amplicons derived from the unspliced and spliced cDNAs were gel excised and submitted for DNA sequence analysis and the resulting data were compared with the control non-spliced DNA template. Comparative sequence analysis showed that the 556 bp RT-PCR product was the result of the group IIA1 intron being spliced out and the joining of the flanking exon segments. Based on a previous study [22] it was expected that the RT-PCR product obtained from a spliced transcript should be 538 bp in length. The additional 18 bp noted was due to a shift of the 5′ splice junction which was 18 nucleotides downstream from the original IBS1 and IBS2 elements. This is probably due to a cryptic/alternate splice site that was utilized in *E*. *coli*. However, the 3′ splice site was found to be consistent with previous studies on this intron in its “native” mitochondrial environment [22].

Similar results were obtained for demonstrating the splicing competency of the group IIB intron RNA extracted from bacterial cells grown in the presence of increasing concentrations of MgCl_2_ (see Material and Method section—*in vivo* RNA splicing assay) yielded a RT-PCR product of 658 bp representing the spliced version of the HEase transcript. However, no splicing was observed when the cells were grown in the absence or in the presence of 1 mM and 20 mM concentrations of external MgCl_2_ (Fig 3B). Sequence analysis of the RT-PCR product revealed that the splicing of this group IIB intron occurred as predicted based on its IBS1 sequence [37] and the splicing followed the conventional intron/splice sites yielding a continuous I-CthI ORF. To demonstrate the splicing competency of group IIA1 and IIB intron in the presence of CoCl_2_ or both MgCl_2_ and CoCl_2_ in the growth media, RNA was extracted from *E*.*coli* BL21 cells containing the constructs I-CthI-[IIA1]-pET28b (+) or I-CthI-[IIB]-pET28b (+). RT-PCR results showed that the bacterial cells grown in either 10 μM of CoCl_2_ or the addition of both 10 μM of CoCl_2_ and 5 mM MgCl_2_ in the growth media did not show any evidence for splicing and only the full length unspliced PCR product was recovered (Fig 3C).

### Alternate splice site for the group IIA1 does not affect I-CthI functionality

Sequence analysis of the cDNA (556 bp) from the group IIA1 intron construct derived transcripts had revealed a cryptic splice site which is 18 nucleotides downstream of the original (native) IBS sequences. The alternate IBS sequences can potentially H-bond with sequences that are near or overlap with the native (original) EBS sequences (S1A Fig). In order to evaluate if the addition of the extra six amino acids could affect the active site or the protein’s tertiary structure, the online program Protein Homology/analogY Recognition Engine V2.0 (PHYRE2) (http://www.sbg.bio.ic.ac.uk/phyre2/html/page.cgi?id+index) [43] was used to map the position of these newly added amino acids onto the predicted tertiary structure of I-CthI. The program showed that the protein is composed of ten alpha helices (39%) and nine beta stands (26%), the confidence key of these regions were found to be 100% when compared to the crystal structure of the HEase I-SmaMI (PDB accession number: c4loxA). The additional six amino acids V, R, R, C, G and Y are located in a linker region of the HEase protein and do not appear to disrupt the active sites (LAGLIDADG motifs) or beta sheets required for making contact with the DNA target site (S1B Fig). The HEase protein derived from the group IIA1 intron containing construct was purified and challenged with its substrate. The enzyme completely linearized the circular substrate plasmid (3.1 kb) within 90 minutes. However, when this protein was challenged with two different non-substrates to test its specificity, even after two hours of incubation at 37°C, the protein failed to cleave the non substrates (S1C Fig). It was also noted that the addition of 10 μM CoCl_2_ in the *in vitro* endonuclease reaction buffer did not inhibit the functionality of the I-CthI HEase and this protein could cleave its substrate in 90 minutes at 37°C. However, when the substrate was incubated just in the presence of 10 μM CoCl_2_ without MgCl_2_ in the endonuclease reaction buffer, the protein did not initiate any cleavage activity (S2 Fig).

### *In vitro* and *in vivo* translation show evidence of HEase protein production under specific [Mg^+2^]

**Fig 4:**
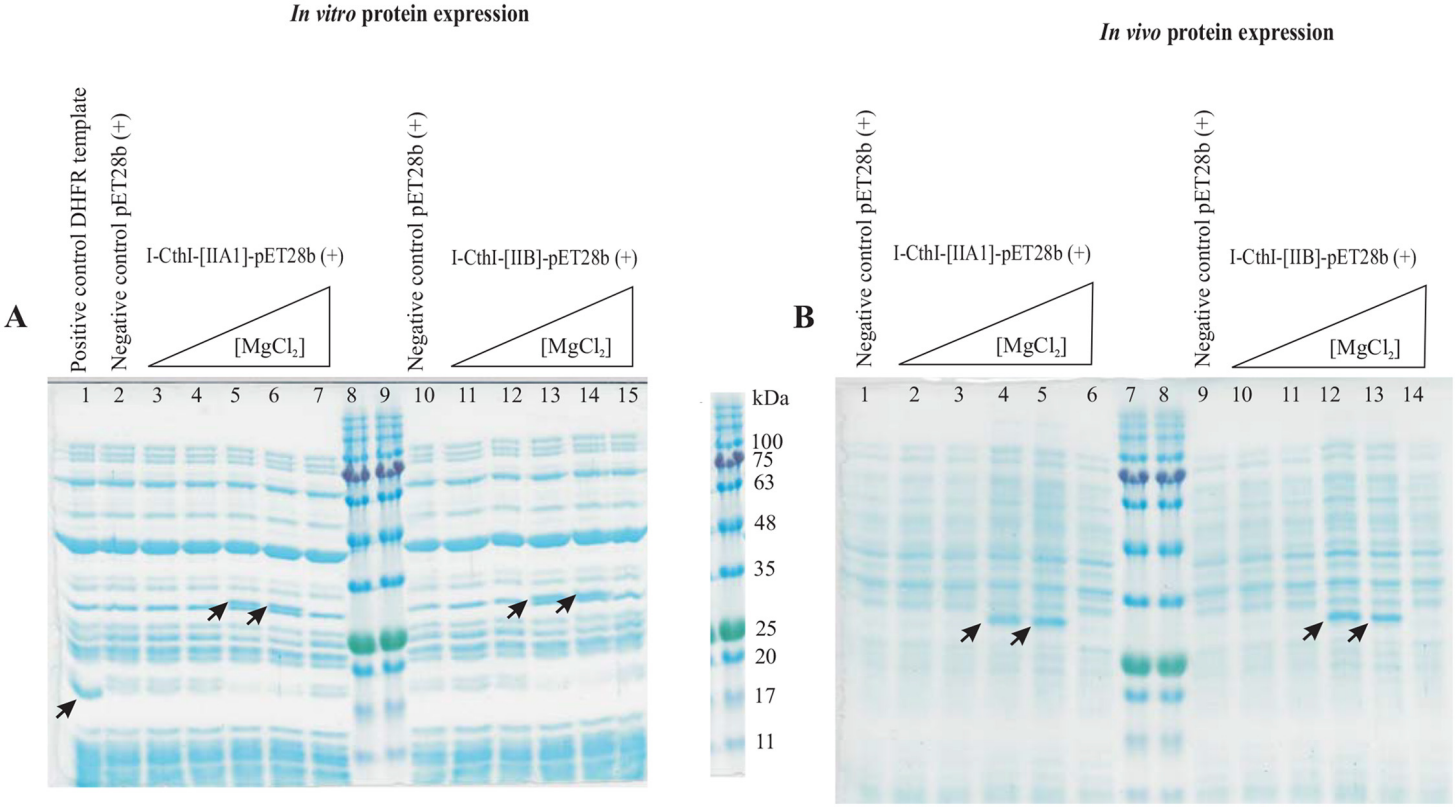
**(A) The effect of MgCl**_**2**_ **on *in vitro* protein expression.** A 12.5% SDS-PAGE showing *in vitro* protein expression for constructs I-CthI-[IIA1]-pET28b (+) [left] and I-CthI-[IIB]-pET28b (+) [right] in the presence of various concentrations of external MgCl_2_ in the culture media. Lane 1 represents the *E*.*coli* dihydrofolate reductase (marked with arrow) when 125 ng/μL was used as the template (positive control) for the PURExpress *In Vitro* Protein Synthesis kit. Lanes 2 and 10 show the *in vitro* protein expression profiles when empty pET28b (+) vectors (without the above constructs) were used as the negative control. Lanes 3 and 11 represent the *in vitro* protein expression profile when RNA (extracted from the culture in the absence of MgCl_2_) was used as the template. Lanes 4 through 7 represent the protein expression profiles when RNA (extracted from the cultures in the presence of 1 mM, 5 mM, 10 mM and 20 mM respectively) was used as the template for the *in vitro* protein synthesis. The expression of the protein (I-CthI) has been marked with arrows. For *in vitro* expression from the I-CthI-[IIB]-pET28b (+) construct, lanes 12 through 15 follow the same order as depicted for the I-CthI-[IIA1]-pET28b (+) construct (i.e. lanes 4–7). Lanes 8 and 9 represent the Blueye prestained protein ladder (FroggaBio, North York, Ontario). **(B) The effect of MgCl**_**2**_ **on *in vivo* protein expression.** A 12.5% SDS-PAGE showing *in vivo* protein expression for constructs I-CthI-[IIA1]-pET28b (+) [left] and I-CthI-[IIB]-pET28b (+) [right] in the presence of various concentrations of external MgCl_2_ in the culture media. Lanes 1 and 9 represent the *in vivo* protein expression profiles from the empty pET28b (+) vector (without the constructs). Lanes 2 through 6 represent the protein expression profiles when I-CthI-[IIA1]-pET28b (+) [BL21] was grown under increasing concentrations of external MgCl_2_ starting from 0 mM, 1 mM, 5 mM, 10 mM and 20 mM. Lane 10 through 14 represent the protein expression profiles when I-CthI-[IIB]-pET28b (+) (BL21) was grown under increasing concentrations of external MgCl_2_. Lanes 10 through 14 follow the same order as for the protein expression profiles when I-CthI-[IIA1]-pET28b (+) [BL21] was grown under increasing concentrations of external MgCl_2_ (i.e. lanes 2–6). The overexpressed I-CthI (migrate at ~29 kDa) has been marked with arrows. Lanes 7 and 8 represent the Blueye prestained protein ladder (FroggaBio, North York, Ontario).

SDS PAGE analysis showed the presence of the protein I-CthI at the desired location (~ 29 kDa) only from *in vitro* translation assays that used RNA extracted from cells grown in the culture media supplemented with either 5 mM or 10 mM MgCl_2_. RNA extracted from cells grown in the absence or at 1 mM and 20 mM MgCl_2_ failed to yield the desired protein in the *in vitro* translation assays. This was observed for both of the tested constructs I-CthI-[IIA1]-pET28b (+) and I-CthI-[IIB]-pET28b (+) when the cells were grown at the same concentrations of MgCl_2_ (Fig 4A).

The codon-optimized HEase ORF (from both constructs) expressed in *E*.*coli* BL21 cells only when the cells were grown in the culture media supplemented with 5 mM or 10 mM MgCl_2_ (Fig 4B). The recovery and purification of the HEase protein was achieved by affinity column chromatography involving Superflow^®^ nickel resin (Qiagen, Toronto). A step up gradient of 25 mM, 50 mM and 100 mM imidazole in the wash buffer was used to remove the background proteins while the pure HEase protein was obtained in the elution buffer supplemented with 250 mM imidazole which was later dialyzed to remove any salts. This purified protein was pooled and concentrated (3 mg/mL) in order to perform the *in vitro* endonuclease assay.

### I-CthI ORF interrupted with either a group IIA1 or IIB introns results in the expression of an active HEase

**Fig 5:**
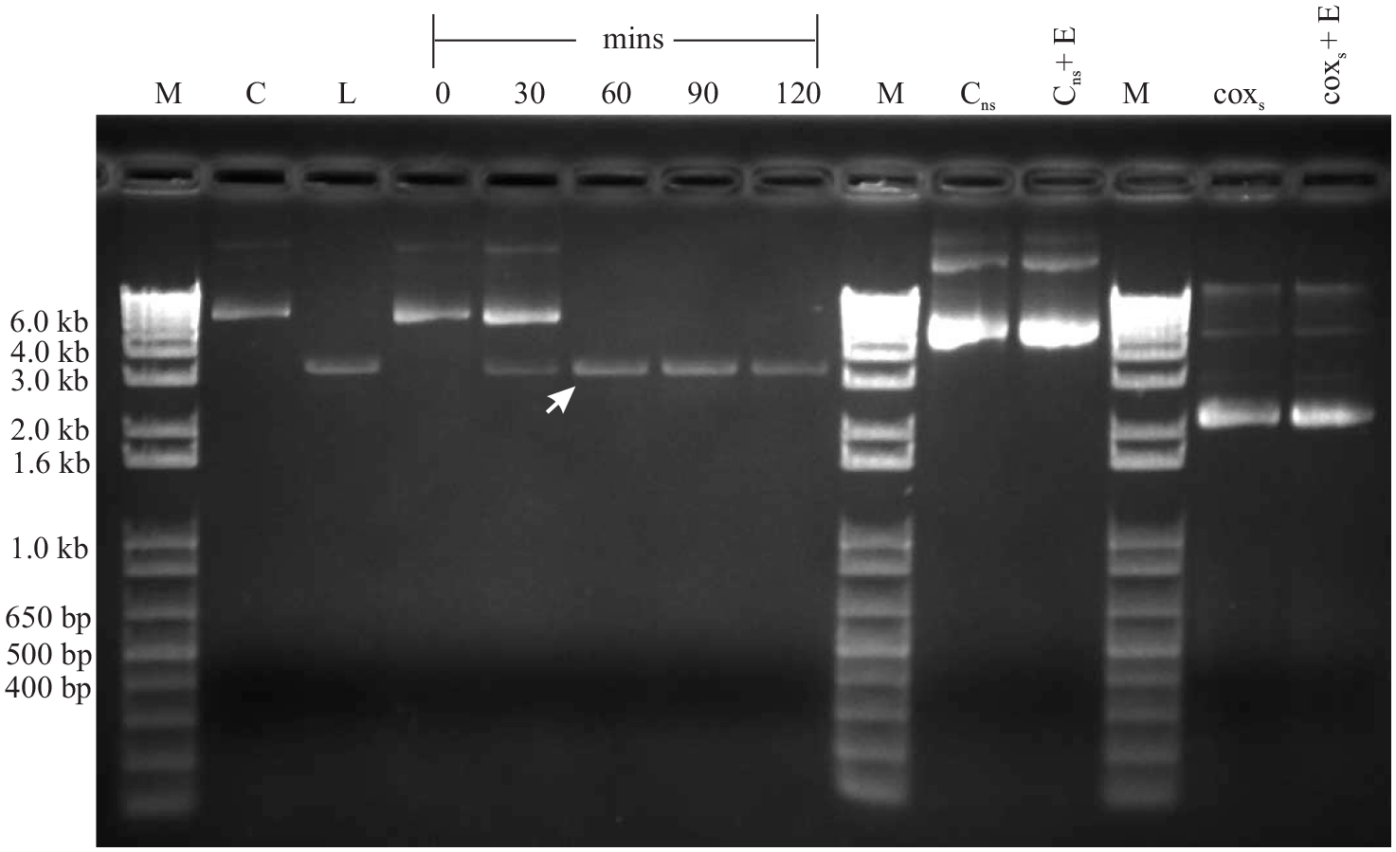
*In vitro* endonuclease assay. A 1% agarose gel showing the *in vitro* endonuclease assay with construct I-CthI-[IIB]-pET28b (+) encoded HEase. Lanes C and L represent uncut substrate plasmid and linearized substrate plasmid (cleaved with BamHI), respectively. Numbers on the top of each lane represent incubation times in minutes at 37°C. For each of the above endonuclease assay, 1 μg of substrate DNA was treated with 8 μL of the purified HEase (2.5 mg/mL). The arrow shows the linearized band at 3.1 kb when the substrate was incubated for 60 minutes. Two negative controls were applied in this assay. C_ns_ and Cox_s_ were used to examine the specificity of this nested intron encoded I-CthI. Cox_s_ is the substrate for another HEase encoded a *cox* gene intron from *Annulohypoxylon stygium* while Cox_s_ + E represents the assay with the same plasmid incubated with purified I-CthI (encoded from the above stated construct) for 120 minutes at 37°C. C_ns_ represents the negative control plasmid previously used [22] in characterizing the I-CthI HEase; this pUC57 based construct contains the *rns* gene plus the mS1247 nested intron. C_ns_ + E represents the negative control plasmid incubated with the same concentration of the HEase for 120 minutes at 37°C. For both the negative controls, no endonuclease activities were observed. Lanes marked with M represent the 1 kb plus DNA ladder (Life Technologies).

The primary objective of this work was to evaluate if group II introns can be utilized as regulatory elements for expressing a mtDNA fungal HEase in *E*. *coli*. Two types of group II introns were utilized, group IIA1 and IIB introns. As previously mentioned *in vitro* endonuclease assays showed that functional HEase were expressed in *E*. *coli* under conditions that favour group II intron splicing. It appears that both types of mitochondrial group II introns can splice in *E*. *coli* under suitable Mg^+2^ concentrations ultimately yielding HEase that can linearize their substrates in one hour at 37°C; and non-substrates were not cleaved even after two hours of incubation indicating that the enzyme is still highly specific for its cleavage site (Fig 5).

### Endonuclease cleavage mapping of HEases derived from ORFs interrupted by group II introns shows cleavage sites have not changed

LAGLIDADG HEases tend to generate cohesive termini by generating staggered cuts with four nucleotide 3′-single stranded overhangs. The T4 DNA polymerase treated and religated I-CthI cleaved substrate plasmid sequences when compared with the sequence of the uncut substrates showed that a 5′-AAGA-3′ segment was removed from the sense strand. The endonuclease cleavage mapping data is shown in S3 Fig. So the cleavage mapping site experiments showed that the intron contained HEases ORFs (containing either the group IIA1 or IIB intron) ultimately allowed for the expression of a functional I-CthI protein that cleaves 8 bp downstream of the mS1247 nested intron insertion site (sense strand) or 4 bp downstream of position S1247 at the antisense strand. These results are in agreement with previous experiments utilizing I-CthI constructs that did not contain introns within the HEase ORF [22]; so the presence of the introns and subsequent RNA processing events in *E*. *coli* did not alter the target site specificity of the HEase.

### *In vivo* endonuclease assays for HEase activity in the presence of MgCl_2_ and/or CoCl_2_

*In vivo* endonuclease assays were performed to evaluate the effect of the addition of either MgCl_2_ and/or CoCl_2_ on the expression and functionality of the I-CthI HEase. The results of the *in vivo* endonuclease assays for HEase activity are depicted in S1, S2 and S3 Tables. Since three technical and two biological replicates (i.e. six independent values) were performed for each of the assay plates, the mean value for the bacterial colony forming units (cfu/mL) are provided along with their respective standard deviations (σ).

First, in order to check whether the protein expressed from I-CthI-[IIA1]-pET28 b (+) construct is functional (i.e. can it cleave) or is toxic to the *E*.*coli* BL21 genome, I-CthI-[IIA1]-pET28b (+) [BL21] was either uninduced or induced with 0.5 mM IPTG in the presence of 5 mM exogenously added MgCl_2_ (S1 Table, left panel). When the uninduced culture was plated on kan plates, the bacterial colony count was calculated 3.2 x 10^10^ cfu/mL, σ = 1.8 x 10^9^ and for the induced culture, the count was 3.0 x 10^10^ cfu/mL, σ = 2.0 x 10^9^. Moreover, a bacterial lawn was observed in the absence of the antibiotic. These data showed that I-CthI is not toxic to *E*. *coli* and it does not appear to have any target specificity within the *E*.*coli* genome. The plate assay results from the negative control pET28b (+) vector cotransformed with Cth-*rns*.pACYC184 substrate are also listed (S1 Table, right panel). The results showed that even in the absence or in the presence of 0.5 mM IPTG, when the cells were plated on cam plates, the bacterial colony count was 4.4 x 10^10^ cfu/mL σ = 2.2 x 10^9^ and 4.2 x 10^10^ cfu/mL σ = 1.8 x 10^9^ respectively. Bacterial lawn was also observed when no antibiotic was applied. These data suggested that the proteins encoded from the empty pET28b (+) vector were not detrimental to the substrate plasmid carrying the cam resistance marker as the cells were viable in the presence of the antibiotic.

**Fig 6:**
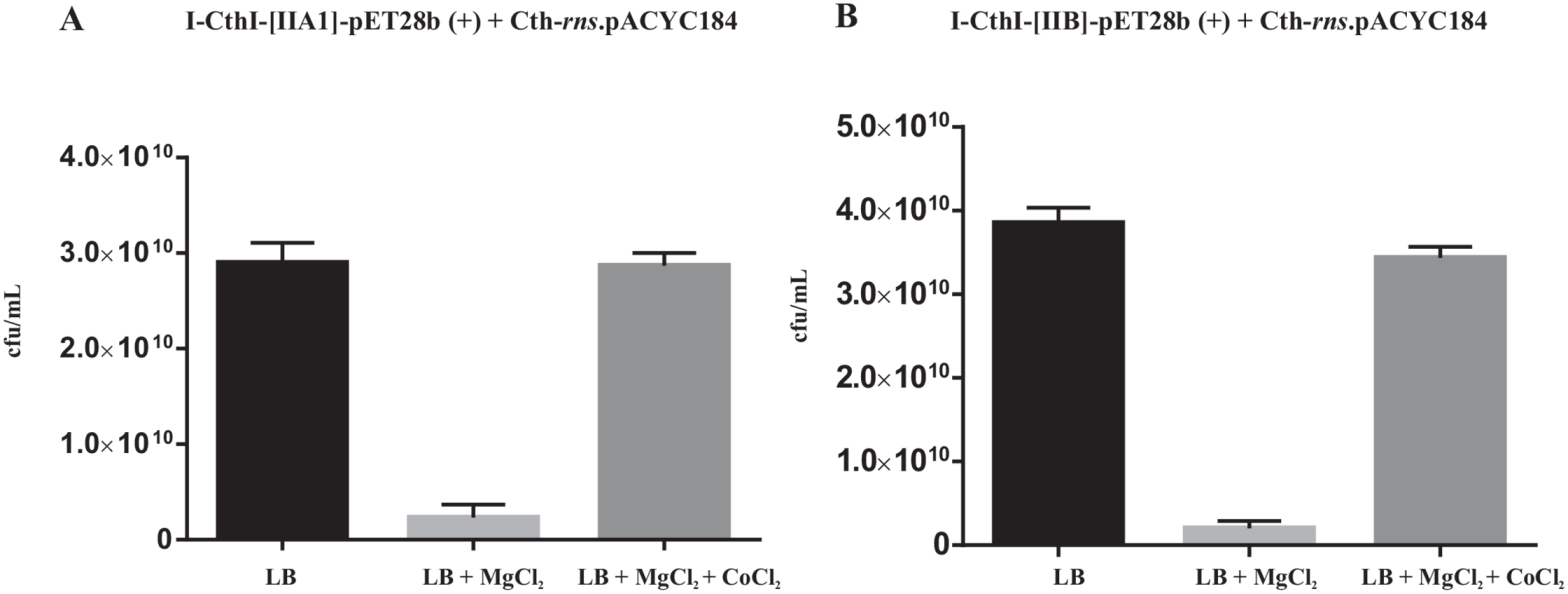
Bar graphs showing the results of the *in vivo* endonuclease assay. Bar graph shown in panel (**A)** represents the Student’s t test performed for assessing the significance of the decrease (p < 0.0001) in the number of viable colonies (cfu/mL) for I-CthI-[IIA1]-pET28b (+) and Cth-*rns*.pACYC184 co-transformed cell lines when grown with the addition of 5 mM MgCl_2_ in the LB media compared to the cells grown in only LB media. Moreover, the graph also shows significant difference (p < 0.0001) with regards to an increase in the number of viable colonies (cfu/mL) when the respective co-transformed cells were grown with the addition of 5 mM MgCl_2_ and 10 μM CoCl_2_ in the LB media compared to the cells grown with 5 mM MgCl_2_ in the LB media. Bar graph shown in panel (**B**) represents the similar results when Student’s t test performed for assessing the significance of the decrease / increase (p < 0.0001) in the number of viable colonies (cfu/mL) for I-CthI-[IIB]-pET28b (+) and Cth-*rns*.pACYC184 co-transformed cell lines grown under the same conditions as described in panel A. Graphpad Prism 6.01 statistical analysis software was used to calculate the Student’s t test and the respective bar graphs were drawn using the same software.

The plate assay results from the *E*. *coli* cells cotransformed with I-CthI-[IIA1]-pET28b (+) and Cth-*rns*.pACYC184 and grown in the absence (LB, left panel) or presence of 5 mM exogenously added MgCl_2_ (LB+Mg^+2^, right panel) are depicted in S2 Table. The results in the left panel show that when the growth media had no exogenously added MgCl_2_, even with or without induction with 0.5 mM IPTG, viable colonies were observed when plated on LB agar-cam plates. The colony count for the uninduced and the induced cultures were 3.0 x 10^10^ cfu/mL σ = 1.3 x 10^9^ and 2.9 x 10^10^ cfu/mL σ = 2.0 x 10^9^ (marked with asterisk in S2 Table, also see S4 Fig, plate 1) respectively indicating that probably absence/inadequate Mg^+2^ concentration in the growth media (irrespective of IPTG induction) did not allow the *in vivo* excision of the internal group II intron thereby not yielding functional HEase. In contrast, in the presence of 0.5 mM IPTG and in the presence of 5 mM MgCl_2_ in the growth media (right panel) allowed for the expression of a functional HEase which probably happened due to the splicing out of the internal intron. This functional protein could have cleaved the target site, thereby degrading the cam resistance substrate plasmid. The bacterial colony count was 2.3 x 10^9^ cfu/mL σ = 1.3 x 10^9^ (marked with asterisk in S2 Table, also see S4 Fig, plate 2). It is worthwhile to mention that there is an approximately 12.6 fold decrease in the bacterial viability when compared to cfu/mL of the cells grown under inducible conditions but in the absence of exogenous MgCl_2_. Student’s t test performed on the cfu/mL indicated significant difference (P value < 0.0001) in the number of viable colonies (decrease) when compared to the cells grown under inducible conditions but in the absence of exogenous MgCl_2_ (Fig 6A).

The results for CoCl_2_ acting as a possible antagonist for the uptake of MgCl_2_ are provided in S3 Table. The left panel of the table shows the results of the *in vivo* endonuclease assay in the presence of only 10 μM of CoCl_2_ in the culture media. This set of assays was performed to rule out the possibility that CoCl_2_ was involved in splicing of the internal intron. Even with or without induction with 0.5 mM IPTG, viable colonies were observed when plated on LB agar-cam plates. The colony count for the uninduced and the induced cultures were 2.2 x 10^10^ cfu/mL σ = 0.9 x 10^9^ and 2.5 x 10^10^ cfu/mL σ = 1.2 x 10^9^ respectively indicating that CoCl_2_ was not involved in facilitating the excision of the group II introns thus yielding a non-functional protein. The right panel shows the results of the *in vivo* endonuclease assay in the presence of both 10 μM of CoCl_2_ and 5 mM MgCl_2_ in the culture media. Indeed, viable cells with colony count of 2.8 x10^10^ cfu/mL, σ = 1.3 x 10^9^ (marked with asterisk in S3 Table, also see S4 Fig, plate 3) were observed on the LB agar cam plates. Student’s t test performed on the cfu/mL indicated significant difference (P value <0.0001) in the number of viable colonies (increase) when the cells were grown with the addition of both 5 mM MgCl_2_ and 10μM CoCl_2_ compared to the cells grown with the addition of only 5 mM MgCl_2_ under inducible conditions (Fig 6A). Assuming that CoCl_2_ antagonizes somehow MgCl_2_ uptake one would predict that under both inductive and non-inductive conditions cells should be viable when plated on LB cam plates as the substrate plasmid carrying the antibiotic resistance marker (cam) was not targeted hence maintained.

The plate assay results from the second cotransformed construct I-CthI-[IIB]-pET28b (+) and Cth-*rns*.pACYC184 are provided in the S4 and S5 Tables, and the images of the LB agar cam plates are presented in S5 Fig, plates 1–3. For this construct 19 fold decrease in the bacterial viability was observed when compared to cfu/mL of the cells grown under inducible conditions but in the absence of exogenous MgCl_2_. Student’s t test performed on the cfu/mL indicated significant difference (p value < 0.0001) in the number of viable colonies (decrease) when the cells were grown under inducible conditions but in the absence of exogenous MgCl_2_ (Fig 6B). Student’s t test performed on the cfu/mL indicated significant difference (p value <0.0001) in the number of viable colonies (increase) when the cells were grown with the addition of both 5 mM MgCl_2_ and 10μM CoCl_2_ compared to the cells grown with the addition of only 5 mM MgCl_2_ under inducible conditions (Fig 6B).

## Discussion

Group II introns by manipulating their EBS elements are currently utilized as genome editing tools in the form of targetrons which can be applied for targeted insertional mutagenesis [44, 45]. Previously we described a mtDNA encoded HEase that is encoded within a group I intron (mS1247) where the intron encoded ORF is disrupted by an ORFless group IIA1 intron [22]. This arrangement hinted at the possibility that the group II intron could be regulatory in nature with regards to the expression of the HEase. Herein we are applying group II introns as regulatory element that allowed for the expression of a fungal mtDNA HEase within *E*. *coli*. Sequences representing either a group IIA1 or a group IIB type intron where inserted into the ORF for the HEase I-CthI at positions that allows for proper intron/exon (i.e. EBS/IBS) interactions so that splicing competent folds could be achieved.

It has been previously shown that an organellar group IIB intron can splice in *E*. *coli* [37], in this study we show that group IIA1 introns also have the potential to splice in *E*. *coli*. It is assumed that group II introns for efficient splicing require intron and/or host encoded factors [25, 26, 46, 47] so this would suggest that within *E*. *coli* factors are available that can be recruited for the removal of the organellar introns that were investigated in this study. In both cases HEase expressed from constructs where the HE ORFs were disrupted by group II introns were active and cut their respective substrates at the expected cleavage sites.

For the group IIB intron the intron/exon junctions based on RT-PCR on RNA extracted from *E*. *coli* were as expected based on previous reports [37]. However, for the group IIA1 intron the intron/exon junction shifted by 18 nucleotides adding 6 amino acid residues to the HEases. These alternate IBS/EBS interactions might be fortuitous but might suggest that this group IIA1 intron splices differently in *E*. *coli* compared to its native environment. Therefore, with regards to designing HEases with an “intron based” regulatory element it is important to evaluate the intron/exon junction in the alternate host environment to ensure HEase functionality/specificity has not been compromised. With regards to I-CthI the altered splicing of the group IIA1 intron added six amino acids to a segment of the protein that apparently did not alter the HEases cleavage specificity or the stability of the protein.

HEases have applications in (a) synthetic biology such as iBrick [48] for the assemble of DNA molecules, (b) as genome editing tools by promoting homologous repair (gene replacements), (c) as a gene targeting tool by promoting mutation inducing non homologous end-joining repair, or (d) as rare cutting enzymes that are part of cloning vectors and cloning strategies [34, 35]. In some instances, such as *in vivo* gene targeting temporal regulation of HEase activity might be desirable in order to minimize nonspecific activity of the enzyme. This study showed that modulating the activity of I-CthI in *E*. *coli* can be accomplished by inserting group II intron sequences into the HEase ORF as splicing of the intron can be stimulated by the addition of Mg^+2^ or antagonized by the addition of Co^+2^. This strategy would have applications in bacterial systems which are more emendable to group II intron splicing unlike eukaryotic cells [48–50]. Group II intron sequences in general are readily available [51] and unlike previous attempts to control HEase activity via redox switches (see PI-SceI [19]) *in vivo* applications are possible.

The Mg^+2^ transport systems in *E*.*coli* have not yet been fully elucidated [52]. In one study, magnesium has been shown to modulate the function of riboswitches by facilitating the ligand-riboswitch interactions e.g. btuB riboswitch from *E*. *coli* [53]. In our study, exogenous Mg^+2^ concentration was evaluated for manipulating intron splicing which allowed for attenuating the expression of a HEase. In the presence of certain concentrations of Mg^+2^ (5 mM or 10 mM) in the growth media the group II introns appeared to splice and thus functional HEases were produced. Magnesium appears to act as a cationic trigger which might enter the *E*. *coli* cells through the magnesium transport systems, raising the intracellular magnesium concentration hence facilitating intron splicing. In order to assess if intron splicing is occurring due to the import of Mg^+2^ in the bacterial cells, CoCl_2_ was used to antagonize the Mg^+2^ effect. Earlier studies have shown that cobaltous ion, at concentrations as low as 10 μM, inhibits the energy-dependent transport of Mg^+2^ into cells of *E*.*coli* [40, 41]. *In vivo* endonuclease assays in the presence of MgCl_2_ and 10 μM of CoCl_2_ showed a reduction in the expression of HEase. This is probably due to Co^+2^ interfering with the entry of magnesium into the cells, leading to Mg^+2^ levels that are not amenable to intron splicing. The failure of the addition of 20 mM MgCl_2_ to stimulate splicing might be an indication that excess Mg^+2^ can interfere with the proper folding of the group II introns [54–56].

Recently the RNA-guided CRISPR-associated (Cas) endonuclease Cas9 has been developed into a genome editing tool [56–60] and it appears well suited for mammalian systems although off-target activity is a concern [61, 62]; Cas9 also appears to be less effective in *E*. *coli* [62–64]. With regards to addressing the off-target activity several methods have been developed to control the nuclease activity of Cas9, such as generating versions of Cas9 that are split into two components and these have been engineered to combine by the addition of a chemical signal such as rapamycin or by blue light irradiation (i.e. a photoactivatable form of Cas9) [65, 66]. Another strategy has been to place an “intein” sequence within Cas9 and the intein has been engineered to splice from the host protein when a ligand (4-hydroxytamoxifen) is added to the media [67]. This ligand-dependent intein is somewhat analogous to our “self-splicing” group II introns that can be promoted to splice at the RNA level when suitable levels of Mg^+2^ are present in the media. One can foresee the application of group II intron sequences as agents that allow for inducible genome editing in cell types that are amendable to support the splicing of these elements and can uptake suitable amounts of Mg^+2^. The ability to antagonize splicing with Co^+2^ provides a “switch like” mechanism where the production of HEase can be stopped or at least attenuated to limit the amount of endonuclease that is produced in a cell and thus potentially avoid nonspecific activities.

In the future with regards to group II intron based “switches” one could achieve even more tighter control by utilizing trans-splicing group II introns. Trans-splicing group II introns (or fragmented group II introns) have been noted in organellar genomes, however it is unknown if these types of introns can function in *E*. *coli* [68, 69]. However, it has been shown that the Ll.LtrB group II intron (including a version where the ORF was deleted) from the Gram-positive bacterium *Lactococcus lactis* can splice in *trans* when fragmented at various locations throughout its structure [70]. Therefore, a HEase ORF could be split and encoded by two compatible plasmids, carrying different selectable markers and different promoters; with one construct bearing the amino terminal part of the HEase ORF plus the 5' segment of a group II intron sequence and the other construct carrying the 3' segment of group II intron sequence plus the carboxyl terminal part of the HEase ORF. Upon expression, these two RNAs can assemble via the intron segments into a tertiary structure that promotes trans-splicing of the intron sequences and thus the exons get ligated together to produce a functional HEase transcript.

The current study is “a proof of principle” that shows that the expression of a gene can be controlled or at least attenuated by the activity of autocatalytic group II intron sequences. The exact nature of Mg^+2^ or Co^+2^ transport from the media into *E*. *coli* is not clear but based on our data we can conclude that manipulation of the concentration of positive cations such as Mg^+2^ and Co^+2^ can influence splicing of heterologous introns within *E*. *coli*. Group II introns could be applied to other heterologous or native proteins that are components of biochemical pathways to allow for temporal control of their expression and possibly promote a shift in metabolic processes. Therefore, in the future group II introns could be a potential tool that can be applied not only to genome editing but also to metabolic engineering [71–73].

## Supporting Information

S1 Fig **(A) Intron and exon binding sites for the mS1247 nested group IIA1 intron.** Watson-Crick base pairing (shown by solid black dots) between the newly discovered cryptic (marked by asterisk sign) splice site sequence (IBS1* and IBS2*) and corresponding exon binding sequences (EBS1* and EBS2*) of the mS1247 internal group IIA1 intron. The original IBS1, IBS2 and EBS1, EBS2 for mS1247 nested intron from *C*. *thermophilum* are indicated. **(B) An *in silico* model for the expressed I-CthI protein.** An *in silico* model for the I-CthI protein derived from the I-CthI-[IIA1]-pET28b (+) construct generated by the PHYRE2 program. The program identified the double motif LAGLIDADG I-SmaMI (PDB: c4loxA) HEase protein as a template for folding I-CthI. Alpha helices and beta sheets along with amino terminal (N) and carboxyl terminal **(C)** have been marked. The LAGLIDADG motifs contribute towards the active site of the enzyme while the beta sheets arrange in a configuration that forms the DNA binding surface. The extra six amino acids (V, R, R, C, G and Y) were not present in any of the active sites of the HEase instead they are located in a linker region between the two beta sheets near the carboxyl terminal of the protein. The linker region showing the extra six amino acids has been magnified for better illustration. The amino acid positions are also mentioned. **(C) *In vitro* endonuclease assay for I-CthI.** A 1% agarose gel showing the *in vitro* endonuclease assay with *C*. *thermophilum* HEase ORF intron containing construct I-CthI-[IIA1]-pET28b (+). Lane C and L represent uncut substrate plasmid and linearized (L) substrate plasmid (cleaved with BamHI), respectively. Numbers on the top of each lane represent incubation time in minutes at 37°C. For each of the above endonuclease assays, 1 μg of substrate DNA was treated with 8 μL of the purified HEase (3 mg/mL). The arrow shows the linearized band at 3.1 kb when the substrate was incubated for 90 minutes. C_ns_ represents the negative control plasmid while C_ns_ + E represents the negative control plasmid incubated with the same concentration of the HEase for 120 minutes at 37°C. Another negative control plasmid (Cox_s_) was used to examine the specificity of this nested intron encoded I-CthI. Cox_s_ is the substrate for another HEase encoded from the intron of the *cox* gene from *Annulohypoxylon stygium* while Cox_s_ + E represent the same plasmid incubated with purified I-CthI (encoded from the above construct) for 120 minutes at 37°C. For both the negative controls, no endonuclease activities were observed. Lane denoted with M represents the 1 kb plus DNA ladder (Life Technologies).
(TIF)

S2 Fig CoCl_2_ does not affect I-CthI endonuclease activity. A 1% agarose gel showing the effect of addition of 10 μM CoCl_2_ during the *in vitro* endonuclease assay with construct I-CthI-[IIA1]-pET28b (+) and I-CthI-[IIB]-pET28b (+) encoded I-CthI HEase. Lane 2 represents the uncut substrate (C) plasmid. Lane 3 shows the endonuclease activity of I-CthI on the substrate plasmid in the presence of 10 μM CoCl_2_ alone in the endonuclease reaction buffer without 10 mM MgCl_2_. Lane 4 represents the linearized substrate (L) when treated with BamHI. Numbers on the top of the lanes (30, 60, 90) represent incubation time in minutes at 37°C. The white arrow shows the linearized band at 3.1 kb. The same order (i.e. lanes 9 through 14) was maintained for the endonuclease activity of the I-CthI HEase derived from I-CthI-[IIB]-pET28b (+) [BL21] construct. Lanes 1, 8 and 15 contain the 1 kb DNA ladder (Life Technologies).
(EPS)

S3 Fig Endonuclease cleavage mapping for HEases derived from ORFs interrupted by group II introns (IIA or IIB). **(A)** The cleavage sites were mapped by comparing uncut substrate with I-CthI treated substrate DNAs. Cleavage by I-CthI generates a staggered cut with 4 nucleotide 3’ overhang in the substrate plasmid at the enzyme’s target site. T4 DNA polymerase was used to blunt the cleaved ends. The religated plasmid was sequenced and compared to the sequence of the untreated substrate plasmid in order to map the cleavage site by scanning for a 4 bp deletion in the T4 DNA polymerase treated cleaved substrate plasmid. **(B)** Schematic representation of the I-CthI cleavage site near the mS1247 intron insertion site. Proposed cleavage sites are indicated by open triangles; and a vertical line represents the intron insertion site. The HEase cleavage site is 8 nt downstream of the intron insertion site with regards to the sense strand or 4 nt downstream with regards to the antisense strand.
(TIF)

S4 Fig Effect of MgCl_2_ on cell viability due to I-CthI activity. Images of LB agar cam plates depicting *in vivo* endonuclease assays performed to evaluate the effect of the addition of either MgCl_2_ and/or CoCl_2_ on the expression and functionality of the I-CthI HEase within cells cotransformed with I-CthI-[IIA1]-pET28b (+) and Cth-*rns*.pACYC184. Two biological and three technical replicates were performed however, one representative from each has been shown. Plate 1, 2 and 3 represent the viable number of colonies when the 100 μL of 10^−6^ cotransformed cells (induced with 0.5 mM IPTG) from 0 mM MgCl_2_, 5 mM MgCl_2_ and 10 μM CoCl_2_ + 5 mM MgCl_2_ in the LB growth media were plated on LB agar plates supplemented with 60 μg/mL cam respectively.
(TIF)

S5 Fig Effect of CoCl_2_ on cell viability due to I-CthI activity. Images of LB agar cam plates depicting *in vivo* endonuclease assays performed to evaluate the effect of the addition of either MgCl_2_ and/or CoCl_2_ on the expression and functionality of the I-CthI HEase in cells cotransformed with I-CthI-[IIB]-pET28b (+) and Cth-*rns*.pACYC184. Two biological and three technical replicates were performed however, one representative from each has been shown. Plate 1, 2 and 3 represent the viable number of colonies when the 100 μL of 10^−6^ cotransformed cells (induced with 0.5 mM IPTG) from 0 mM MgCl_2_, 5 mM MgCl_2_ and 10 μM CoCl_2_ + 5 mM MgCl_2_ in the LB growth media were plated on LB agar plates supplemented with 60 μg/mL cam respectively.
(TIF)

S1 Table *In vivo* activity of I-CthI expressed from I-CthI-[IIA1]-pET28b (+). *In vivo* endonuclease activity of the HEase expressed from the I-CthI-[IIA1]-pET28b (+) [BL21] construct and pET28b (+) and challenged with the substrate plasmid Cth-*rns*.pACYC184 [BL21]; results reported cfu/mL. Three technical and two biological replicates were performed for each of the constructs.The numbers represent the mean of six independent cfu/mL. Standard deviations are also indicated for each of the above observations.
(DOCX)

S2 Table Effect of 5 mM MgCl_2_ on the *in vivo* activity of I-CthI-[IIA1]. *In vivo* endonuclease activity of I-CthI-[IIA1]-pET28b (+) + Cth-*rns*.pACYC184 [BL21] cotransformed constructs presented in cfu/mL. This table presents the plate assay results of the above construct under different conditions, one is without added MgCl_2_ and the other is with the addition of 5 mM MgCl_2_. Three technical and two biological replicates were performed for each of the constructs and the numbers represent the mean of six independent cfu/mL. Standard deviations are also indicated for each of the above observations. * mark on specific boxes indicates that the images of the plates (Plate D) are provided in the S4 Fig.
(DOCX)

S3 Table Effect of CoCl_2_ on the *in vivo* activity of I-CthI-[IIA1]. Cobalt chloride antagonism on the possible uptake of magnesium in *E*.*coli* cells as shown by the *in vivo* endonuclease activity of the HEase expressed from I-CthI-[IIA1]-pET28b (+) and challenged witht the substrate plasmid Cth-*rns*.pACYC184 [BL21]; results are presented in cfu/mL.This table shows the plate assay results of the above constructs under different conditions, one is with the addition of 10 μM CoCl_2_ and the other is with the addition of both 10 μM CoCl_2_ and 5 mM MgCl_2_ in the LB media.Three technical and two biological replicates were performed for each of the constructs and the numbers represent the mean of six independent cfu/mL. Standard deviations are also indicated for each of the above observations. * mark on specific boxes indicates that the images of the plates (Plate D) are provided in the S4 Fig.
(DOCX)

S4 Table *In vivo* activity of I-CthI expressed from I-CthI-[IIB]-pET28b (+). *In vivo* endonuclease assay showing the HEase activity as demonstated in cells that were cotransformed with I-CthI-[IIB]-pET28b (+) and Cth-*rns*.pACYC184 [BL21]; results are reported in cfu/mL. The plate assay results of the above construct under different conditions, one is without added MgCl_2_ and the other is with addition of 5 mM MgCl_2_. Standard deviations are also indicated for each of the above observations. * mark on specific boxes (Plate D) indicates that the images of the plates (Plate D) are provided in the S5 Fig.
(DOCX)

S5 Table *In vivo* activity of I-CthI-[IIB] in the presence of CoCl_2_. Cobalt chloride antagonism on the possible uptake of magnesium in *E*.*coli* cells during the *in vivo* HEase endonuclease assay in cells cotransformed with I-CthI-[IIB]-pET28b (+) and Cth-*rns*.pACYC184 [BL21]; results reported in cfu/mL. This table presents the plate assay results of the above construct under different conditions with the addition of either exogeneous CoCl_2_ (10 μM) or 10 μM CoCl_2_ and 5 mM MgCl_2_ in the LB media. Standard deviations are also indicated for each of the above results. * mark on specific boxes indicates that the images of the plates (Plate D) are provided in the S5 Fig.
(DOCX)
