# Supplementary figures and images for: Using Group II Introns for Attenuating the In Vitro and In Vivo Expression of a Homing Endonuclease
Source: PLoS One. 2016 Feb 24;11(2):e0150097. doi: 10.1371/journal.pone.0150097 (PMC4801052; doi:10.1371/journal.pone.0150097)

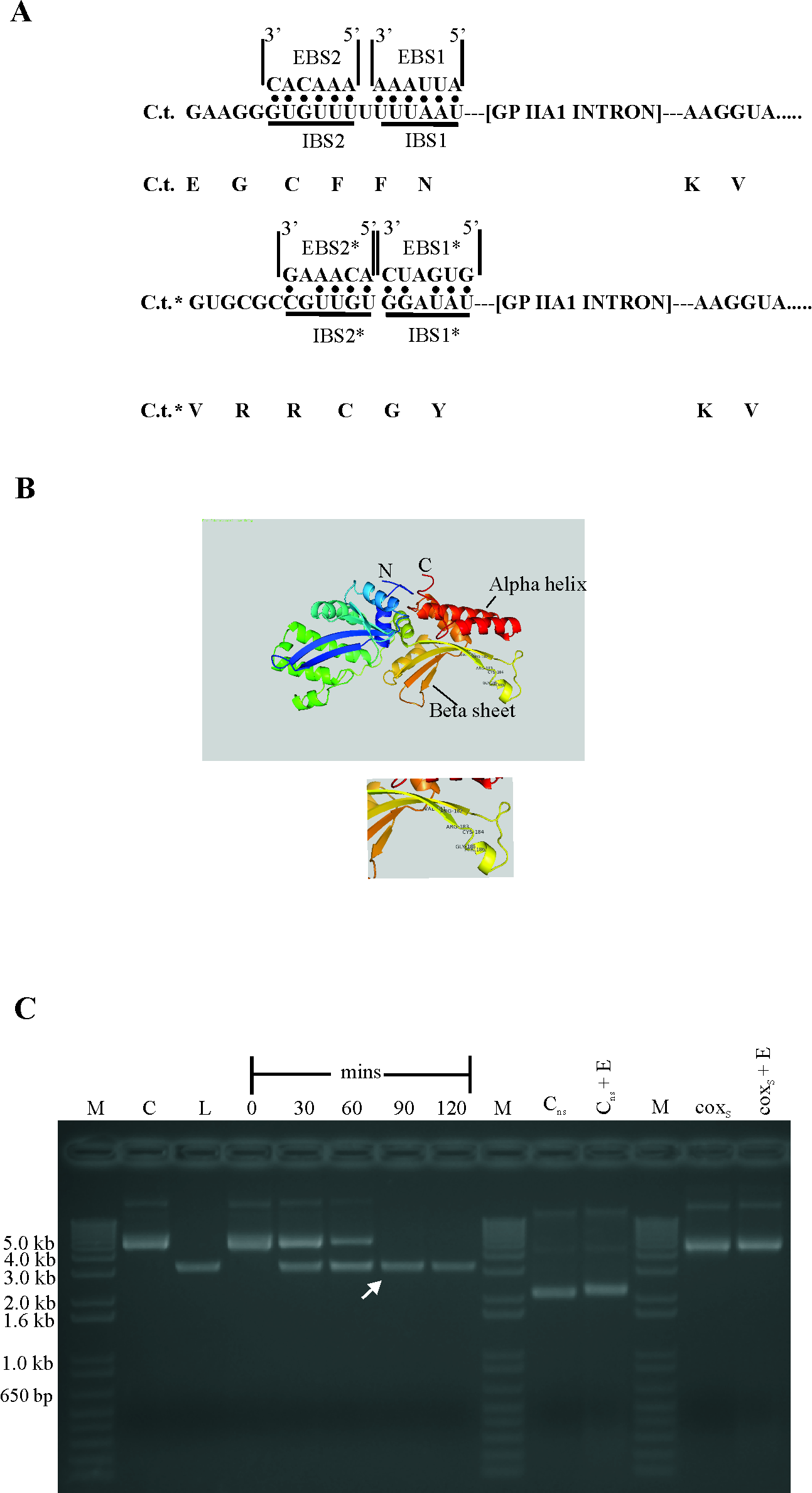

Supplement: S1 Fig — (A) Intron and exon binding sites for the mS1247 nested group IIA1 intron. Watson-Crick base pairing (shown by solid black dots) between the newly discovered cryptic (marked by asterisk sign) splice site sequence (IBS1* and IBS2*) and corresponding exon binding sequences (EBS1* and EBS2*) of the mS1247 internal group IIA1 intron. The original IBS1, IBS2 and EBS1, EBS2 for mS1247 nested intron from C. thermophilum are indicated. (B) An in silico model for the expressed I-CthI protein. An in silico model for the I-CthI protein derived from the I-CthI-[IIA1]-pET28b (+) construct generated by the PHYRE2 program. The program identified the double motif LAGLIDADG I-SmaMI (PDB: c4loxA) HEase protein as a template for folding I-CthI. Alpha helices and beta sheets along with amino terminal (N) and carboxyl terminal (C) have been marked. The LAGLIDADG motifs contribute towards the active site of the enzyme while the beta sheets arrange in a configuration that forms the DNA binding surface. The extra six amino acids (V, R, R, C, G and Y) were not present in any of the active sites of the HEase instead they are located in a linker region between the two beta sheets near the carboxyl terminal of the protein. The linker region showing the extra six amino acids has been magnified for better illustration. The amino acid positions are also mentioned. (C) In vitro endonuclease assay for I-CthI. A 1% agarose gel showing the in vitro endonuclease assay with C. thermophilum HEase ORF intron containing construct I-CthI-[IIA1]-pET28b (+). Lane C and L represent uncut substrate plasmid and linearized (L) substrate plasmid (cleaved with BamHI), respectively. Numbers on the top of each lane represent incubation time in minutes at 37°C. For each of the above endonuclease assays, 1 μg of substrate DNA was treated with 8 μL of the purified HEase (3 mg/mL). The arrow shows the linearized band at 3.1 kb when the substrate was incubated for 90 minutes. Cns represents the negative control plasmid w [file pone.0150097.s001.tif]

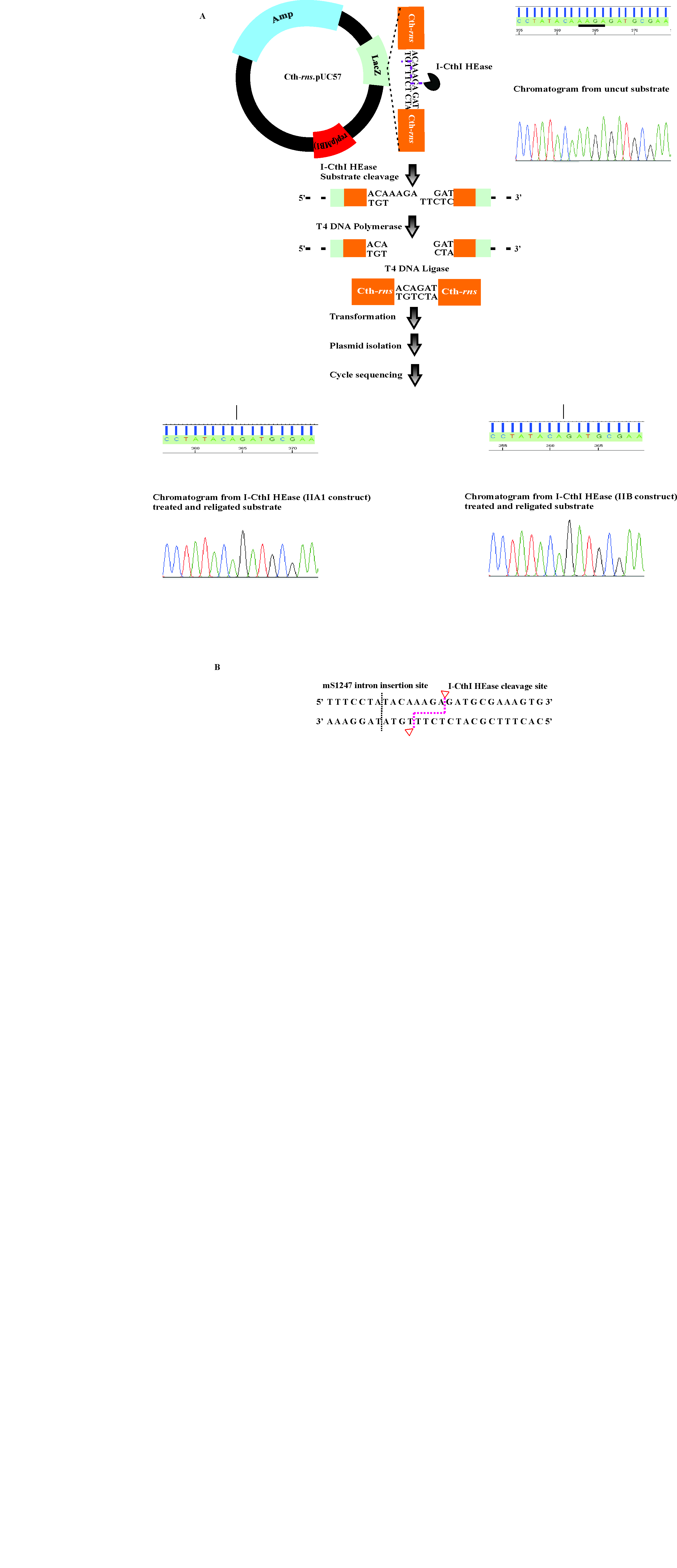

Supplement: S3 Fig — (A) The cleavage sites were mapped by comparing uncut substrate with I-CthI treated substrate DNAs. Cleavage by I-CthI generates a staggered cut with 4 nucleotide 3’ overhang in the substrate plasmid at the enzyme’s target site. T4 DNA polymerase was used to blunt the cleaved ends. The religated plasmid was sequenced and compared to the sequence of the untreated substrate plasmid in order to map the cleavage site by scanning for a 4 bp deletion in the T4 DNA polymerase treated cleaved substrate plasmid. (B) Schematic representation of the I-CthI cleavage site near the mS1247 intron insertion site. Proposed cleavage sites are indicated by open triangles; and a vertical line represents the intron insertion site. The HEase cleavage site is 8 nt downstream of the intron insertion site with regards to the sense strand or 4 nt downstream with regards to the antisense strand. (TIF) [file pone.0150097.s003.tif]

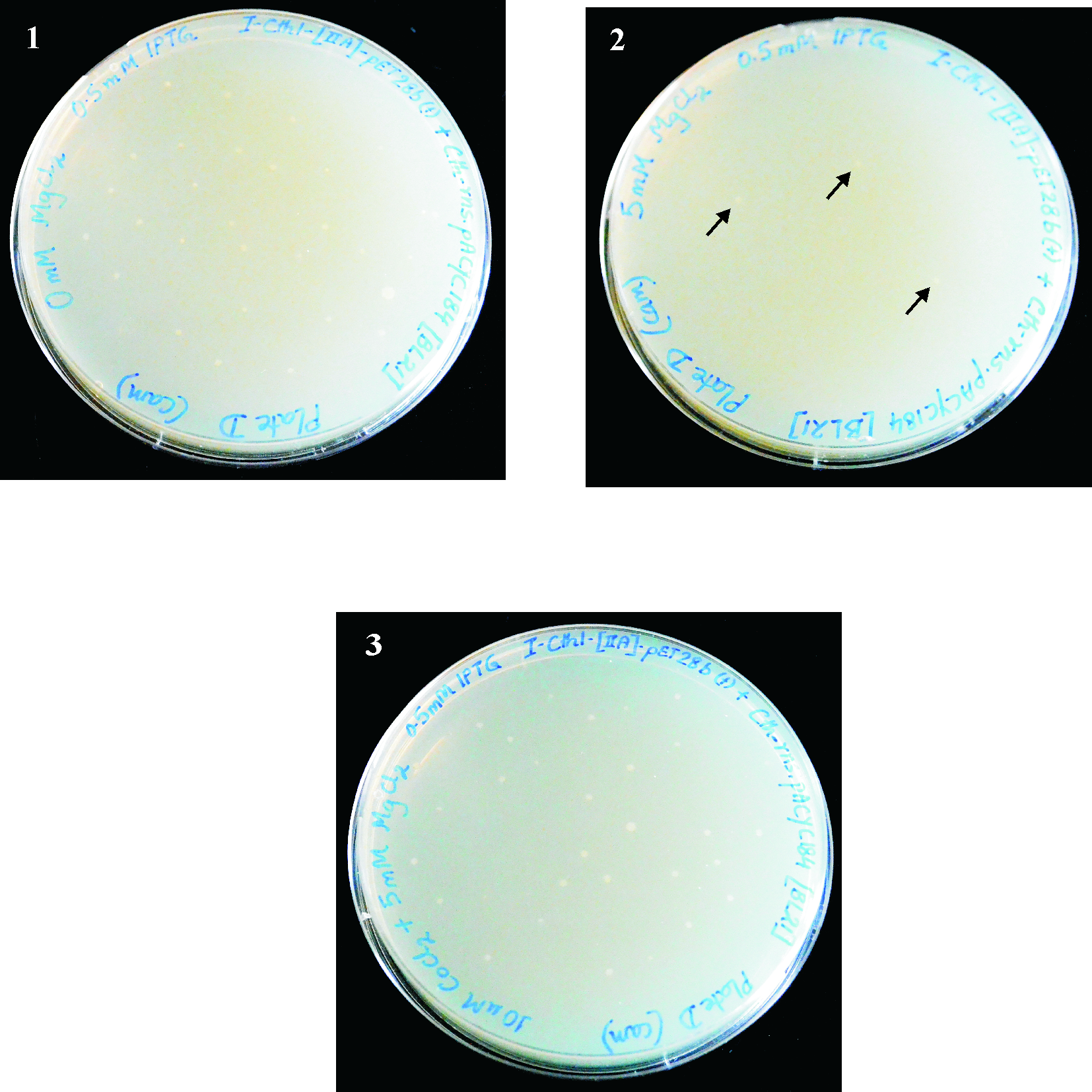

Supplement: S4 Fig — Images of LB agar cam plates depicting in vivo endonuclease assays performed to evaluate the effect of the addition of either MgCl2 and/or CoCl2 on the expression and functionality of the I-CthI HEase within cells cotransformed with I-CthI-[IIA1]-pET28b (+) and Cth-rns.pACYC184. Two biological and three technical replicates were performed however, one representative from each has been shown. Plate 1, 2 and 3 represent the viable number of colonies when the 100 μL of 10−6 cotransformed cells (induced with 0.5 mM IPTG) from 0 mM MgCl2, 5 mM MgCl2 and 10 μM CoCl2 + 5 mM MgCl2 in the LB growth media were plated on LB agar plates supplemented with 60 μg/mL cam respectively. (TIF) [file pone.0150097.s004.tif]

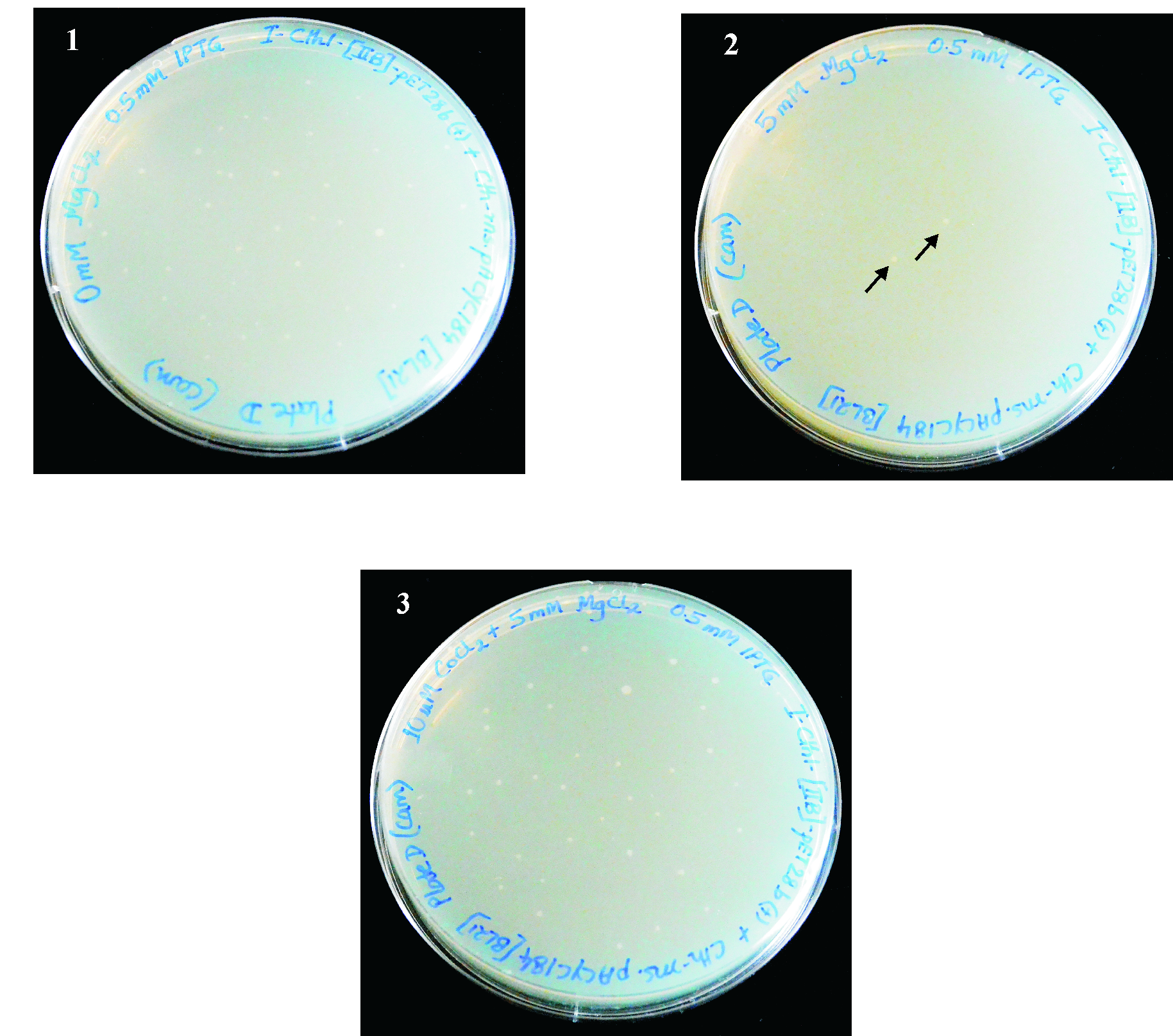

Supplement: S5 Fig — Images of LB agar cam plates depicting in vivo endonuclease assays performed to evaluate the effect of the addition of either MgCl2 and/or CoCl2 on the expression and functionality of the I-CthI HEase in cells cotransformed with I-CthI-[IIB]-pET28b (+) and Cth-rns.pACYC184. Two biological and three technical replicates were performed however, one representative from each has been shown. Plate 1, 2 and 3 represent the viable number of colonies when the 100 μL of 10−6 cotransformed cells (induced with 0.5 mM IPTG) from 0 mM MgCl2, 5 mM MgCl2 and 10 μM CoCl2 + 5 mM MgCl2 in the LB growth media were plated on LB agar plates supplemented with 60 μg/mL cam respectively. (TIF) [file pone.0150097.s005.tif]
