# Supplementary material for: Using Group II Introns for Attenuating the In Vitro and In Vivo Expression of a Homing Endonuclease
Source: PLoS One. 2016 Feb 24;11(2):e0150097. doi: 10.1371/journal.pone.0150097 (PMC4801052; doi:10.1371/journal.pone.0150097)
Supplement: S1 Table — In vivo endonuclease activity of the HEase expressed from the I-CthI-[IIA1]-pET28b (+) [BL21] construct and pET28b (+) and challenged with the substrate plasmid Cth-rns.pACYC184 [BL21]; results reported cfu/mL. Three technical and two biological replicates were performed for each of the constructs.The numbers represent the mean of six independent cfu/mL. Standard deviations are also indicated for each of the above observations. (DOCX) [file pone.0150097.s006.docx]

| **Plate assay (two biological and three technical replicates)** | **I-CthI-[IIA1]-pET28b(+)**  **[BL21]** | **pET28b(+) +**  **Cth-*rns*.pACYC184**  **[BL21]** |
| --- | --- | --- |
| Plate ‘A’  No antibiotic | Bacterial lawn observed | Bacterial lawn observed |
| Plate ‘B’  (kan + cam) | Not applicable | 3.0 x 10^10^ cfu/mL σ = 0.8 x 10^9^ |
| Plate ‘C’ No induction  (cam) 5 mM MgCl_2_ | 3.2 x 10^10^ cfu/mL σ = 1.8 x 10^9^ | 4.4 x 10^10^ cfu/mL σ = 2.2 x 10^9^ |
| Plate ‘D’ 0.5 mM IPTG  (cam) 5 mM MgCl_2_ | 3.0 x 10^10^ cfu/mL σ = 2.0 x 10^9^ | 4.2 x 10^10^ cfu/mL σ = 1.8 x 10^9^ |

**S1 Table. *In vivo* endonuclease activity of I-CthI-[IIA1]-pET28b (+) [BL21] construct and**

**pET28b (+) + Cth-*rns*.pACYC184 [BL21] construct presented in cfu/mL.**
