# Supplementary material for: Using Group II Introns for Attenuating the In Vitro and In Vivo Expression of a Homing Endonuclease
Source: PLoS One. 2016 Feb 24;11(2):e0150097. doi: 10.1371/journal.pone.0150097 (PMC4801052; doi:10.1371/journal.pone.0150097)
Supplement: S2 Table — In vivo endonuclease activity of I-CthI-[IIA1]-pET28b (+) + Cth-rns.pACYC184 [BL21] cotransformed constructs presented in cfu/mL. This table presents the plate assay results of the above construct under different conditions, one is without added MgCl2 and the other is with the addition of 5 mM MgCl2. Three technical and two biological replicates were performed for each of the constructs and the numbers represent the mean of six independent cfu/mL. Standard deviations are also indicated for each of the above observations. * mark on specific boxes indicates that the images of the plates (Plate D) are provided in the S4 Fig. (DOCX) [file pone.0150097.s007.docx]

|  | **0 mM MgCl_2_ in LB media** | **5 mM MgCl_2_ in LB media** |
| --- | --- | --- |
| **Plate assay (two biological and three technical replicates)** | **I-CthI-[IIA1]-pET28b (+) +**  **Cth-*rns*.pACYC184**  **[BL21]** | **I-CthI-[IIA1]-pET28b (+) +**  **Cth-*rns*.pACYC184**  **[BL21]** |
| Plate ‘A’  No antibiotic | Bacterial lawn observed | Bacterial lawn observed |
| Plate ‘B’  (kan + cam) | 3.3 x 10^10^ cfu/mL σ = 3.7 x10^9^ | 3.1 x 10^10^ cfu/mL σ = 1.4 x 10^9^ |
| Plate ‘C’  No induction  (cam) | 3.0 x 10^10^ cfu/mL σ = 1.3 x 10^9^ | 2.8 x 10^10^ cfu/mL σ = 1.2 x 10^9^ |
| Plate ‘D’  0.5 mM IPTG  (cam) | 2.9 x 10^10^ cfu/mL σ = 2.0 x 10^9^  ***** | 2.3 x 10^9^ cfu/mL σ = 1.3 x 10^9^  ***** |

**S2 Table. *In vivo* endonuclease activity of I-CthI-[IIA1]-pET28b (+) + Cth-*rns*.pACYC184**

**[BL21] cotransformed constructs presented in cfu/mL.**
