# Supplementary material for: Using Group II Introns for Attenuating the In Vitro and In Vivo Expression of a Homing Endonuclease
Source: PLoS One. 2016 Feb 24;11(2):e0150097. doi: 10.1371/journal.pone.0150097 (PMC4801052; doi:10.1371/journal.pone.0150097)
Supplement: S3 Table — Cobalt chloride antagonism on the possible uptake of magnesium in E.coli cells as shown by the in vivo endonuclease activity of the HEase expressed from I-CthI-[IIA1]-pET28b (+) and challenged witht the substrate plasmid Cth-rns.pACYC184 [BL21]; results are presented in cfu/mL.This table shows the plate assay results of the above constructs under different conditions, one is with the addition of 10 μM CoCl2 and the other is with the addition of both 10 μM CoCl2 and 5 mM MgCl2 in the LB media.Three technical and two biological replicates were performed for each of the constructs and the numbers represent the mean of six independent cfu/mL. Standard deviations are also indicated for each of the above observations. * mark on specific boxes indicates that the images of the plates (Plate D) are provided in the S4 Fig. (DOCX) [file pone.0150097.s008.docx]

|  | **10 µM CoCl_2_ in LB media** | **10 µM CoCl_2_ + 5 mM MgCl_2_ in LB media** |
| --- | --- | --- |
| **Plate assay (two biological and three technical replicates)** | **I-CthI-[IIA1]-pET28b (+) +**  **Cth-*rns*.pACYC184**  **[BL21]** | **I-CthI-[IIA1]-pET28b (+) +**  **Cth-*rns*.pACYC184**  **[BL21]** |
| Plate ‘A’  No antibiotic | Bacterial lawn observed | Bacterial lawn observed |
| Plate ‘B’  (kan + cam) | 2.9 x 10^10^ cfu/mL σ = 2.6 x 10^9^ | 2.3 x 10^10^ cfu/mL σ = 1.5 x 10^9^ |
| Plate ‘C’  No induction  (cam) | 2.2 x 10^10^ cfu/mL σ = 0.9 x 10^9^ | 2.1 x 10^10^ cfu/mL σ = 1.6 x 10^9^ |
| Plate ‘D’  0.5 mM IPTG  (cam) | 2.5 x 10^10^  cfu/mL σ = 1.2 x 10^9^ | 2.8 x 10^10^ cfu/mL σ = 1.3 x 10^9^  ***** |

**S3 Table. Cobalt chloride antagonism on possible uptake of magnesium in *E.coli* cells using**

***in vivo* endonuclease activity of I-CthI-[IIA1]-pET28b (+) + Cth-*rns*.pACYC184 [BL21]**

**cotransformed constructs presented in cfu/mL.**
