# Supplementary material for: Using Group II Introns for Attenuating the In Vitro and In Vivo Expression of a Homing Endonuclease
Source: PLoS One. 2016 Feb 24;11(2):e0150097. doi: 10.1371/journal.pone.0150097 (PMC4801052; doi:10.1371/journal.pone.0150097)
Supplement: S4 Table — In vivo endonuclease assay showing the HEase activity as demonstated in cells that were cotransformed with I-CthI-[IIB]-pET28b (+) and Cth-rns.pACYC184 [BL21]; results are reported in cfu/mL. The plate assay results of the above construct under different conditions, one is without added MgCl2 and the other is with addition of 5 mM MgCl2. Standard deviations are also indicated for each of the above observations. * mark on specific boxes (Plate D) indicates that the images of the plates (Plate D) are provided in the S5 Fig. (DOCX) [file pone.0150097.s009.docx]

|  |  |  |
| --- | --- | --- |
|  | **0 mM MgCl_2_ in LB media**  **(LB)** | **5 mM MgCl_2_ in LB media**  **(LB+Mg^+2^)** |
| **Plate assay (two biological and three technical replicates)** | **I-CthI-[IIB]-pET28b(+) +**  **Cth-*rns*.pACYC184 [BL21]** | **I-CthI-[IIB]-pET28b(+) +**  **Cth-*rns*.pACYC184 [BL21]** |
| Plate ‘A’  No antibiotic | Bacterial lawn observed | Bacterial lawn observed |
| Plate ‘B’  (kan + cam) | 4.2 x 10^10^ cfu/mL σ = 1.5 x10^9^ | 4.3 x 10^10^ cfu/mL σ = 1.1 x10^9^ |
| Plate ‘C’  No induction  (cam) | 3.5 x 10^10^ cfu/mL σ = 2.8 x10^9^ | 3.1 x 10^10^ cfu/mL σ = 1.3 x10^9^ |
| Plate ‘D’  0.5 mM IPTG  (cam) | 3.8 x 10^10^ cfu/mL σ = 1.8 x10^9^  ***** | 2.0 x 10^9^ cfu/mL σ = 0.8 x 10^9^ |

**S4 Table.** ***In vivo* endonuclease assay showing the HEase activity as demonstated in cells**

**that were contronsformed with I-CthI-[IIB]-pET28b (+) and Cth-*rns*.pACYC184 [BL21]**

**reported in cfu/mL.**
