# Supplementary material for: Using Group II Introns for Attenuating the In Vitro and In Vivo Expression of a Homing Endonuclease
Source: PLoS One. 2016 Feb 24;11(2):e0150097. doi: 10.1371/journal.pone.0150097 (PMC4801052; doi:10.1371/journal.pone.0150097)
Supplement: S5 Table — Cobalt chloride antagonism on the possible uptake of magnesium in E.coli cells during the in vivo HEase endonuclease assay in cells cotransformed with I-CthI-[IIB]-pET28b (+) and Cth-rns.pACYC184 [BL21]; results reported in cfu/mL. This table presents the plate assay results of the above construct under different conditions with the addition of either exogeneous CoCl2 (10 μM) or 10 μM CoCl2 and 5 mM MgCl2 in the LB media. Standard deviations are also indicated for each of the above results. * mark on specific boxes indicates that the images of the plates (Plate D) are provided in the S5 Fig. (DOCX) [file pone.0150097.s010.docx]

|  | **10 μM CoCl_2_ in LB media** | **10 μM CoCl_2_ + 5 mM MgCl_2_ in LB media** |
| --- | --- | --- |
| **Plate assay (two biological and three technical replicates)** | **I-CthI-[IIB]-pET28b (+) +**  **Cth-*rns*.pACYC184 [BL21]** | **I-CthI-[IIB]-pET28b (+) +**  **Cth-*rns*.pACYC184 [BL21]** |
| Plate ‘A’  No antibiotic | Bacterial lawn observed | Bacterial lawn observed |
| Plate ‘B’  (kan + cam) | 2.9 x 10^10^ cfu/mL σ = 2.2 x 10^9^ | 3.2 x 10^10^ cfu/mL σ = 2.1 x 10^9^ |
| Plate ‘C’  No induction  (cam) | 3.6 x 10^10^ cfu/mL σ = 1.7 x 10^9^ | 3.6 x 10^10^ cfu/mL σ = 1.2 x 10^9^ |
| Plate ‘D’  0.5 mM IPTG  (cam) | 3.2 x 10^10^ cfu/mL σ = 2.4 x 10^9^ | 3.4 x 10^10^ cfu/mL σ = 1.3 x 10^9^  ***** |

**S5 Table. Cobalt chloride antagonism on possible uptake of magnesium in *E.coli* cells using**

***in vivo* HEase endonuclease activity in cells cotransformed with I-CthI-[IIB]-pET28b (+)**

**and Cth-*rns*.pACYC184 [BL21] reported in cfu/mL.**
